# Photo-affinity labeling (PAL) in chemical proteomics: a handy tool to investigate protein-protein interactions (PPIs)

**DOI:** 10.1186/s12953-017-0123-3

**Published:** 2017-06-24

**Authors:** Dhiraj P. Murale, Seong Cheol Hong, Md. Mamunul Haque, Jun-Seok Lee

**Affiliations:** 1 Molecular Recognition Research Center, Korea Institute of Science and Technology, 39-1 Hawolgok-dong, Seoul, 136-791 Republic of Korea; 2 Department of Biological Chemistry, KIST-School UST, 39-1 Hawolgok-dong, Seoul, 136-791 Republic of Korea

**Keywords:** Photo-affinity probe, Protein-protein interaction, Quantitative proteomics, Benzophenone, Aryl azide, Diazirine

## Abstract

Protein-protein interactions (PPIs) trigger a wide range of biological signaling pathways that are crucial for biomedical research and drug discovery. Various techniques have been used to study specific proteins, including affinity chromatography, activity-based probes, affinity-based probes and photo-affinity labeling (PAL). PAL has become one of the most powerful strategies to study PPIs. Traditional photocrosslinkers are used in PAL, including benzophenone, aryl azide, and diazirine. Upon photoirradiation, these photocrosslinkers (Pls) generate highly reactive species that react with adjacent molecules, resulting in a direct covalent modification. This review introduces recent examples of chemical proteomics study using PAL for PPIs.

## Background

The visualization and monitoring of specific proteins without disturbing their biological function is a major challenge in chemical biology [1, 2]. To achieve the goals of this study, i.e., the localization and trafficking of a protein of interest (POI), researchers commonly use fluorescent labeling. This technique of genetically encoding fluorescent proteins (FPs) is broadly applied because of its intrinsic specificity [3, 4]. However, this method has some limitations, specifically the size of the green fluorescent protein (GFP) (ca. 30 kDa), which is sufficient to disturb the function and localization of the POI, and the fact that FPs can oligomerize [5]. As an alternative to this technique, chemical tags have been developed for the POI. The chemical tag method is similar to FP tagging. Different types of chemical tags have been developed to reduce the size of the tag. These chemical tags include fluorogenic bisarsenic tags, such as green fluorescent FlAsH and red fluorescent ReAsH tags [6, 7], as well as Halo-tag [8], SNAP-tag [9], CLIP-tag [10], BirA-tag [11, 12], APEX (enhanced ascorbate peroxidase) tag [13], TMP-tag [14, 15], His-tag [16–18]. A powerful alternative strategy to tagging is utilizing in situ photo-affinity labeling (PAL). PAL, or photocrosslinking, is a unique and emergent technique to study protein-protein interactions in the complex proteome. Upon photoirradiation, a photocrosslinking functional group generates highly reactive species that react with adjacent molecules, resulting in direct covalent modification (Table 1) [19–38]. Since PAL can capture noncovalent interaction partners spatio-selectively, photocrosslinking agents have emerged as a critical tool to study PPIs. Despite the significance, few photocrosslinkers are currently available, including benzophenone (BP), aryl azide (AA), and diazirine (DA).

**Table 1:** Major reviews in the field of PAL

Sr. No.	Title	Journal	Authors and brief description
1	Benzophenone Photophores in Biochemistry	Biochemistry, 1994, 33(19), 5661–5673.	Glenn D. Prestwich et al. Here they have shown the detail study of the benzophenone photochemistry. The main points discussed are, overview of the BP photochemistry, and biochemical applications of tethered BPs, Site-directed photolabelling with polypeptides containing amino acids 4-benzoylphenylalanine and related amino acids and photo-crosslinking with heterobifunctional cross-linking agents.
2	Recent Trends in Photoaffinity Labeling	Angew. Chem. Int. Ed. Engl. 1995, 34. 1296–1312.	Maurice Goeldner et al. have talked about the ligand-receptor interactions.
3	Benzophenone Photoprobes for Phosphoinositides, Peptides and Drugs	Photochemistry and Photobiology, 1997, 65(2), 222–234.	Glenn D. Prestwich et al. have discussed BP photoprobes for Phosphoinositides, Peptides and Drugs. The main headlines include: BP and BP-like photosystems, Photochemical and design considerations, Drugs, substrates and inhibitors, Peptides, nucleotides and nucleosides, Phosphoinositides.
4	Recent Progress in Diazirine-Based Photoaffinity Labeling	Eur. J. Org. Chem. 2008, 2513–2523.	Makoto Hashimoto et al. In this review authors mostly give emphasis on the PAL of diazirines mostly up to 2008.
5	Photocrosslinkers illuminate interactions in living cells	Mol. BioSyst. 2008, 4, 473–480.	Jennifer J. Kohler et al. here authors summarized the technology of cellular incorporation of photo-crosslinking amino acids and sugars becomes routine, to analyze crosslinked complexes
6	Target Identification by Diazirine Photo-Cross-Linking and Click Chemistry	Curr. Protoc. Chem. Biol., 2009, 1, 55.	Jack Taunton et al. In this book chapter authors have given insight into the development of diazirine bases PAL.
7	Proteome-wide detection of phospholipid-protein interactions in mitochondria by photocrosslinking and click chemistry	Mol. BioSyst., 2010, 6, 1751–1759.	Anton I. P. M. de Kroon et al. Here they summarize phospholipid-protein interactions in mitochondria by photocrosslinking and click chemistry.
8	Probing small molecule-protein interactions: A new perspective for functional proteomics	Journal of Proteomics, 2011, 75, 100–115.	Mathias Dreger et al. have summarized, probe designs, workflows, and published applications of the three dominant approaches in the field, namely affinity pull-down, activity-based protein profiling, and Capture Compound Mass Spectrometry.
9	Aliphatic Diazirines as Photoaffinity Probes for Proteins: Recent Developments	Chem. Rev. 2011, 111, 4405–4417.	Joydip Das gave the detail summary in the development of aliphatic diazirines.
10	Diazirine based photoaffinity labeling	Bioorg. Med. Chem. 2012, 20, 554–570.	M. Meijler et al. reviewed recent advances in the use of diazirines in photoaffinity labeling till 2012.
11	Recent Advances in Target Characterization and Identification by Photoaffinity Probes	Molecules, 2013, 18, 10425–10451.	Sang J. Chung et al. In this review authors have summarized most of the photoaffinity probes till 2013.
12	Photo-induced covalent cross-linking for the analysis of biomolecular interactions	Chem. Soc. Rev., 2013, 42, 3289–3301.	Andrew J. Wilson et al. In this review authors have summarized wide range of PAL functionalities involving the covalent cross-linking of biomolecules with the affinity tags.
13	From noncovalent to covalent bonds: a paradigm shift in target protein identification	Mol. BioSyst., 2013, 9, 544–550.	S B Park et al. have talked about different techniques to identify the target. These techniques include, Affinity-based target identification, Chemo-reactive group-based target identification, Photo reactive group-based target identification and FITGE-based target identification.
14	Photoactivatable Lipid Probes for Studying Biomembranes by Photoaffinity Labeling	Chem. Rev. 2013, 113, 7880–7929.	Ling Peng et al. have summarized Lipid Probes with Different Reactive Species for Photolabelling.
15	Photocrosslinking approaches to interactome mapping	Current Opinion in Chemical Biology 2013, 17, 90–101	Jennifer J Kohler et al. here authors have discussed the application of cell-based photocrosslinking to the study of specific problems in immune cell signaling, transcription, membrane protein dynamics, nucleocytoplasmic transport, and chaperone-assisted protein folding.
16	Diazirine-based multifunctional photo-probes for Affinity-based elucidation of protein-ligand interaction	Heterocycles 2014, 89 (12), 2697–2727.	Yasumaru Hatanaka et al. have reviewed reflects recent achievements in the chemistry and biological use of the diazirine based PAL reagents.
17	Photoaffinity labeling in target and binding-site identification	Future Med. Chem. 2015, 7(2), 159–183.	Ian Collins et al. have summarized the principles of PAL including probe design and experimental techniques for in vitro and live cell investigations.
18	Photoaffinity Probes for Identification of Carbohydrate-Binding Proteins	Asian J. Org. Chem. 2015, 4, 116–126.	Kaori Sakurai has mentioned the PAL for identification carbohydrate-binding proteins.
19	Genetically Encoded Photocrosslinkers for Identifying and Mapping Protein-Protein Interactions in Living Cells	IUBMB life, 2016, 68(11), 879–886.	Peng R. Chen et al. have reviewed photo-affinity unnatural amino acids that can be site-specifically incorporated into a protein of interest to covalently trap non-covalent PPIs under living conditions.
20	The Life of Pi Star: Exploring the Exciting and Forbidden Worlds of the Benzophenone Photophore	Chem. Rev. 2016, 116, 15284–15398.	Gyorgy Dormán et al. have reviewed, the “forbidden” (transitions) and excitation-activated world of photoinduced covalent attachment of BP photophores.

## Mode of action of PAL

**Fig. 1:**
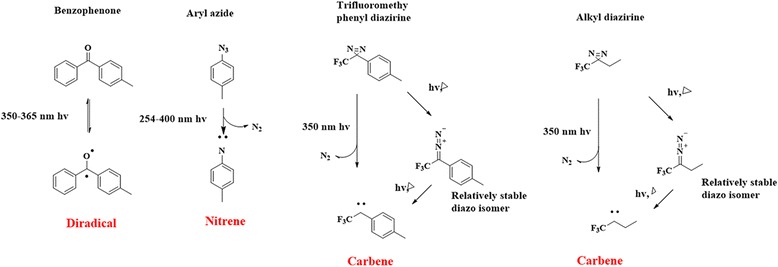
Mode of action of different photocrosslinkers

PAL was developed by Westheimer et al. in 1962 [39]. Since its development, different types of photocrosslinkers have emerged as potential photocrosslinkers. These photocrosslinkers are mainly divided into three photoreactive groups: BPs, DAs, and AAs. Upon photoirradiation, these groups generate reactive intermediates to establish a covalent modification with the POI (Fig. 1).

**Fig. 2:**
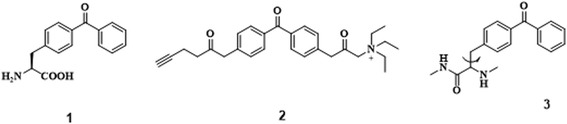
Genetically encoded amino acid p-benzoyl-L-phenylalanine (Bpa) based photo-probes

## Benzophenone

BP was introduced as a photocrosslinker in the 1970s and has since become the most popular photocrosslinker because it is more selective and has high affinity towards methionine. BP is converted into an active diradical upon activation by relatively long (350–365 nm) wavelengths. Early work demonstrated that BP was more suitable for biological applications than other simple aryl ketones (e.g., acetophenones) because the conditions required for photolysis are less damaging to the protein primary structure. BP generates a triplet ketyl biradical that can react with protein functional groups via a sequential abstraction–recombination mechanism.

## Aryl azide

AAs crosslink through a reactive species called nitrene, which is formed by a loss of N_2_ upon photoirradiation with wavelengths ranging between 254 and 400 nm. Nitrene can react with neighboring C–H and heteroatom–H bonds, forming a new covalently bonded product. AAs are chemically stable and has superior photophysical properties than its corresponding alkyl and acyl partners.

## Diazirine

Trifluoromethyl phenyl DAs and alkyl DAs can both generate the reactive species called carbene via loss of N_2_ upon photoirradiation at 350 nm. This active species is very reactive and can be inserted into neighboring C–H or heteroatom–H bonds to form a covalent adduct. Additionally, the generated carbene has a typical half-life in the nanosecond range and can react very quickly. DAs and their diazo isomers both have the capacity to generate the reactive species carbene via the irreversible loss of N_2_.

Among these three photocrosslinkers, BP has the discrete property of repeated photoactivation to form diradicals, but same are not found in the case of the conversion of AAs to nitrenes and DAs to carbenes. This could be the main reason for the increased crosslinking efficiency of BP compared with AA and DA by prolonged UV irradiation.

## Benzophenone-based probes to study PPI

BP was first introduced by Printz et al. in 1974 as an effective functional group for PAL, wherein they presented the ability of BP to photocrosslink with glycine [40].

To study PPI in vitro and in vivo, Peter G. Schultz et al. [41, 42] developed photo-affinity-based genetically encoded amino acids (Fig. 2). They selected BP as the photocrosslinker as it is supposed to be the most useful PAL group in biology. They described the pair of aminoacyl-tRNA synthetase and tRNA, which can be used to incorporate p-benzoyl-L-phenylalanine into proteins in Escherichia coli in response to the amber codon, TAG. This unnatural amino acid was easily incorporated into the dimeric protein glutathione S-transferase with high translational efficiency and fidelity. Upon photoirradiation, efficient crosslinking was observed in >50% of the protein subunits. This technology was proved to be useful to study PPI in vitro and in vivo. A similar approach was proposed by Jason W. Chin et al. [43], who demonstrated facile and site-specific incorporation of the photocrosslinking amino acid pBpa into proteins of any length. Stuart Licht et al. [44] developed an activity-based protein profiling probe (ABPP) for the nicotinic acetylcholine receptor. They designed the probe as a candidate ABPP probe, named BPyneTEA (BP-alkyne-triethylammonium). This probe has the ability to bind to open or closed nAChRs for state-dependent binding and photolabeling of nAChRs along with BP for photoirradiation. This Bpa-based technology was further studied by Angela Wittelsberger’s group [45], who demonstrated that distance restraints based on photo-affinity crosslinking using Bpa (p-benzoylphenylalanine) must be applied to ligand-receptor systems with full knowledge of the limitations and potential drawbacks. They suggested that a distance restraint of at least 10 A° is vital due to the characteristic properties, including its size, physicochemical properties, and conformational flexibility. The Bpa-based technology should be applied to investigate ligand-receptor systems to gain insight into general landmarks and key contact regions.

**Fig. 3:**
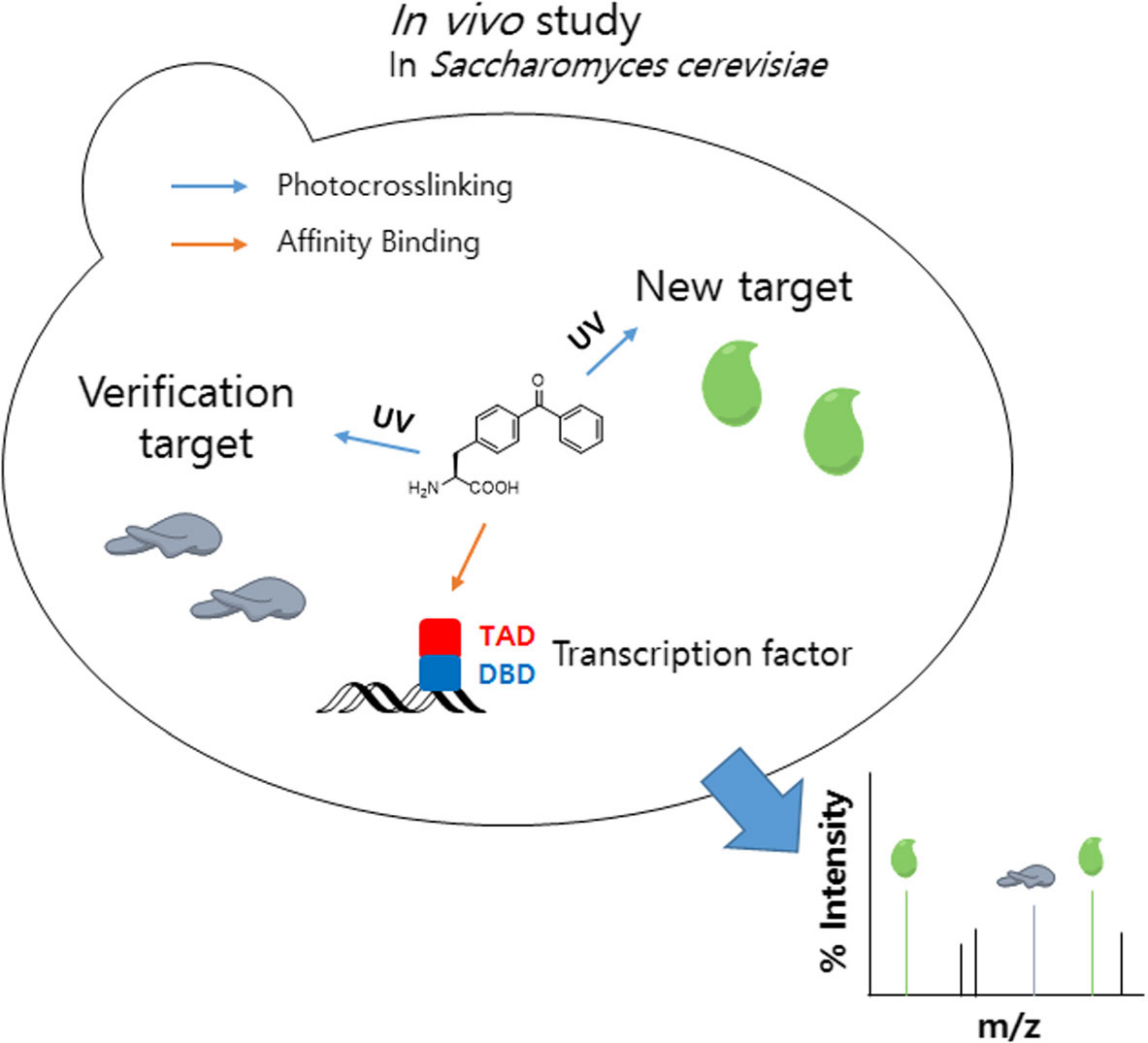
An in vivo covalent chemical capture and mass spectrometric-based approach for the identification of the cellular targets of transcriptional activators

In other studies, Anna K. Mapp et al. [46] used Bpa photocrosslinking for in vivo covalent chemical capture and LC-MS/MS analysis to trap PPIs of transcriptional activators in a cellular environment and to identify the binding partners in an unbiased manner. They presented the discovery of enzymatic targets of transcriptional activators via in vivo covalent chemical capture. The network of activator PPIs that underpin transcription initiation has not been thoroughly investigated, specifically in the cellular context (Fig. 3). This is due to the transient nature of these contacts and the low abundance of the participants. The prototypical activators Gal4 and VP16, which target the Snf1 (AMPK) kinase complex via direct interactions with both the core enzymatic subunit Snf1 and the exchangeable subunit Gal83, were discovered by this approach. Further, the method was used in live yeast to capture the Gal4-Snf1 interaction at the Gal1 promoter using a tandem reversible formaldehyde and irreversible covalent chemical capture approach (TRIC).

**Fig. 4:**
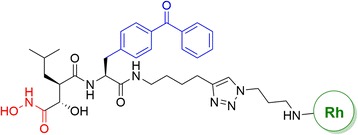
Design of an MP-directed activity-based probe, HxBP-Rh

Another approach to studying PAL is activity-based protein profiling (ABPP). Benjamin F. Cravatt et al. [47] developed the ABPP approach to target metalloproteases (MPs). The key point of their success was the incorporation of hydroxamate and BP groups into the chemical probes (Fig. 4). Hydroxamate was used because it has an affinity towards the zinc atom in the MP active site, and BP was used for the covalent interactions.

**Fig. 5:**
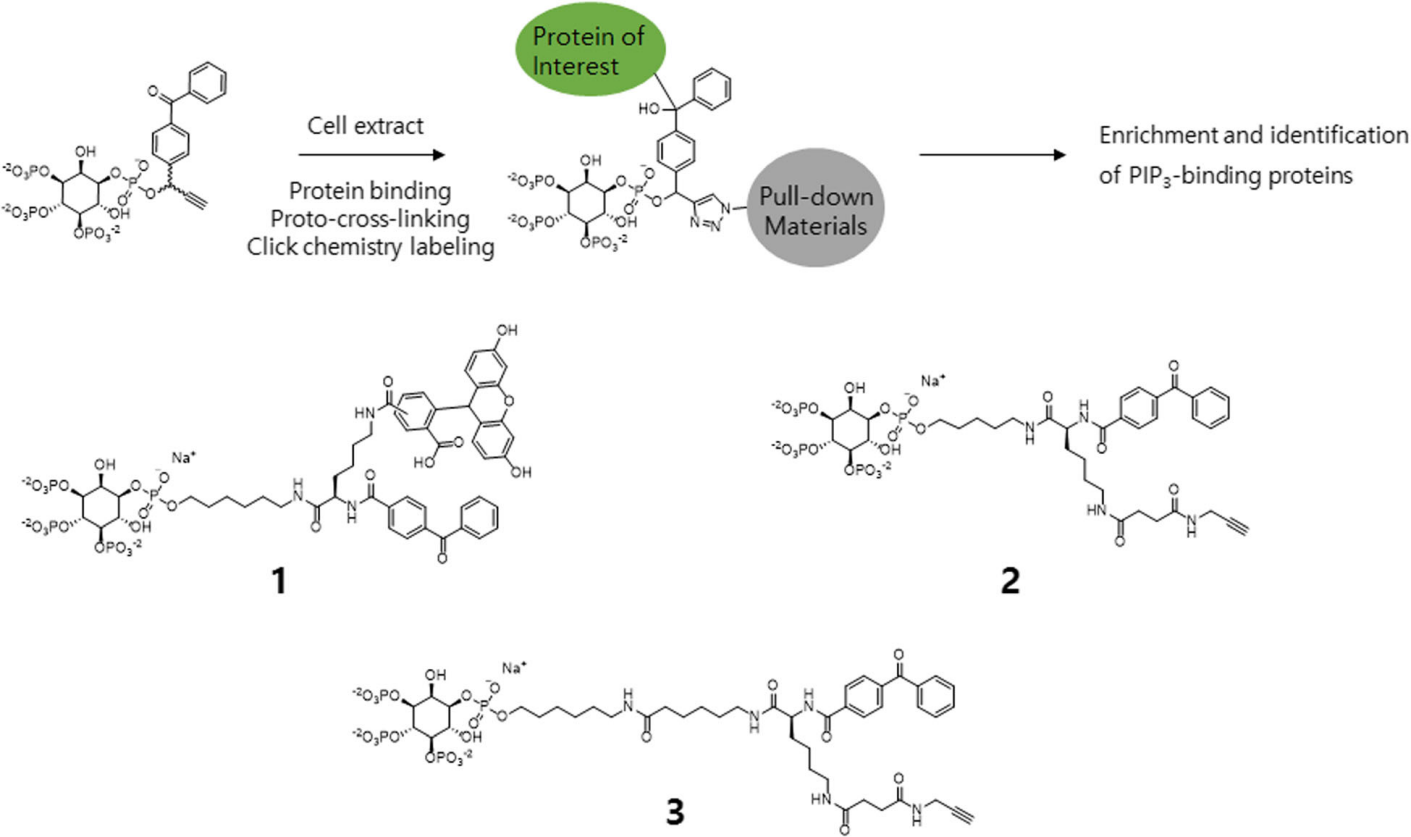
Mode of action and the designs of bifunctional PIP_n_

A similar ABPP approach was used by Michael D. Best et al. [48], who described the use of phosphatidylinositol 3,4,5-trisphosphate activity probes (with BP) for the photolabeling and identification of protein-binding partners (Fig. 5). The activity-based probes include a binding moiety (PIPn headgroup), linked to a Y-shaped lysine linker containing both a photo-affinity group (BP) and a secondary tag with either fluorescein (fluorescence) or an alkyne for bioorthogonal reaction. Additionally, they studied secondary tags either by direct attachment of a fluorescent dye for fluorescence detection or by using alkyne tag click chemistry. First, they described the design and synthesis of multiple probes with different reporter tags that were used to investigate probe-labeled proteins. Next, they performed primary labeling studies using purified protein, the PH domain of Akt, where the labeling of the target was confirmed by in-gel detection. In addition, they added different chain length linkers; the result of photo-affinity labeling led to differences in protein labeling, indicating that a shorter linker was more effective. Finally, proteomic labeling studies were performed using cell extracts; and in-gel detection was used to detect labeled proteins, which were characterized using post-labeling with biotin, affinity chromatography, and mass spectrometry. These studies yielded a total of 265 binding proteins, including both known and novel candidates.

**Fig. 6:**
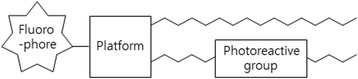
General structure of the lipid tools

Common amino acids have also been modified for PAL. In this regard, the Line Bourel-Bonnet group designed and synthesized a novel class of fluorescent and photosensitive lipid tools with a common amino acid scaffold functionalized by three groups: (i) a first fatty acid chain grafted to a photoactivatable BP moiety (fatty acid BP, FABP), (ii) a second fatty acid chain to ensure anchoring to a half-bilayer or hydrophobic environment, and (iii) a fluorescent carboxytetramethylrhodamine headgroup (CTMR) [49] to detect the photolabelled compound (Fig. 6). The newly designed lipid tools have many advantages, including the ease of synthesis, and the fatty acid chains gave the stability, as well as a hydrophobic environment. Additionally, they are stable in membranes due to their double fatty acid chain structure. Upon photoactivation, BP undergoes covalent interaction with the immediate environment in the membrane. Finally, the CTMR headgroup (fluorophore) enables detection and monitoring of the crosslinking reaction products. Overall, the authors provided a new, robust and efficient tool to study and identify hydrophobic proteins.

**Fig. 7:**
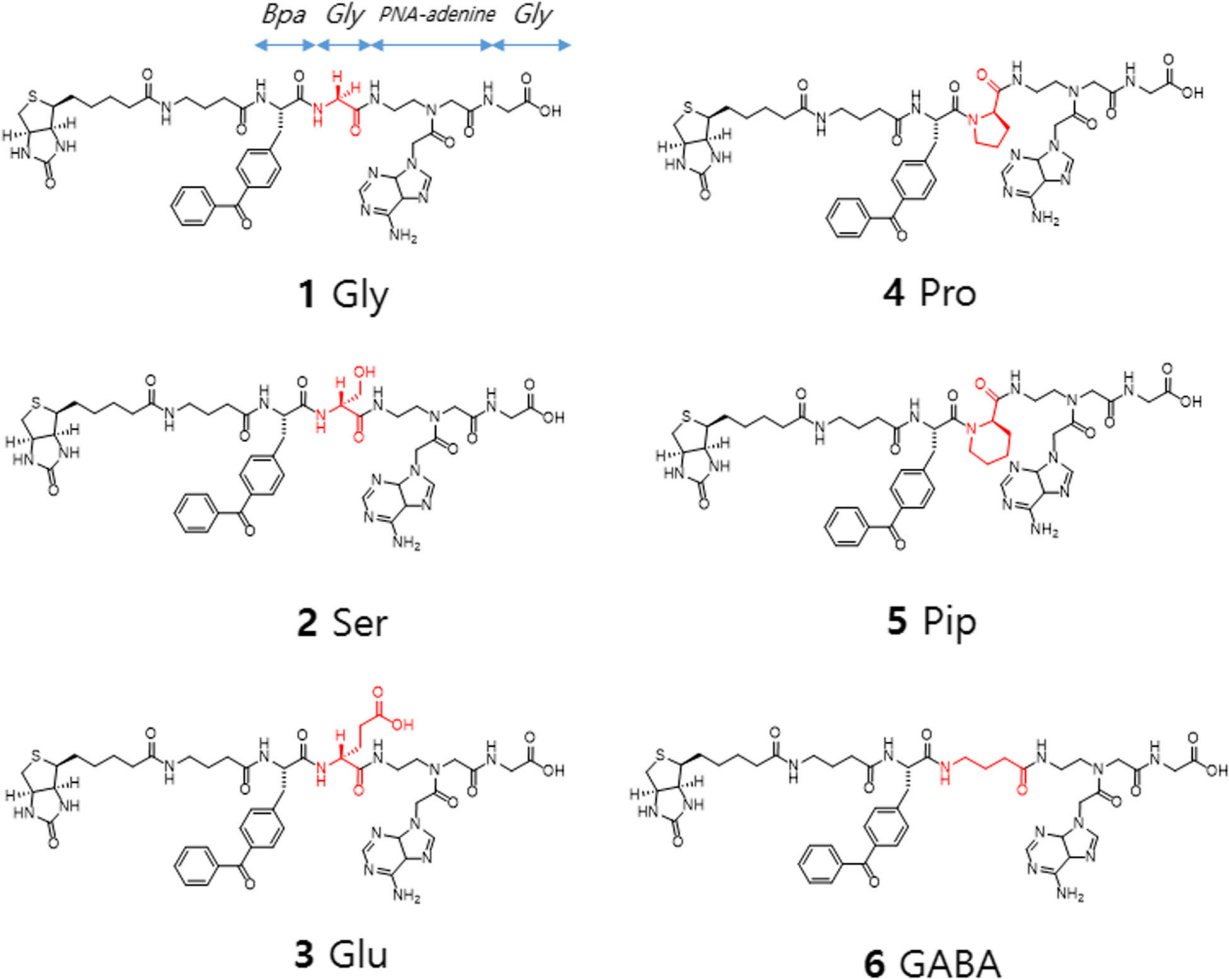
Structures of newly synthesized Lck photoligands

To obtain details about a proteome, the structure-activity relationship (SAR) plays a vital role. Akira Kawamura et al. [50] conducted an SAR study of BP photo ligands for Lck kinase (Fig. 7), in which different photo ligands were compared based on varying target-binding affinity and conformational flexibility. The authors conducted this SAR study to address the issue of photocrosslinking of the target binding because, in many cases, when a photoprobe binds to its target, photocrosslinking does not necessarily occur. This is due to the lack of target-binding affinity and conformational flexibility of the photo ligand. As a result of this study, they found that photolabeling efficiency does not depend upon the kinase inhibitory potency but is dependent on the conformational flexibility of photo ligands. The labeling efficiency can be easily improved by a slight increase in conformational flexibility of the BP photo probes.

**Fig. 8:**
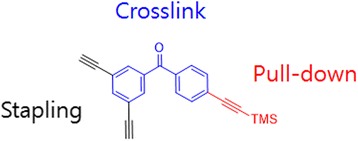
Structure of a novel multifunctional benzophenone linker for photo-crosslinking motif and peptide stapling reagent

To improve the specificity of photocrosslinking, David R. Spring et al. [51] designed and synthesized a novel multifunctional BP linker for a photocrosslinking motif and a peptide stapling reagent (Fig. 8). They used the double-click reaction to attach BP to the peptide via a staple linker instead of modifying the peptide sequence using a photocrosslinking amino acid. They also used a p53-derived peptide that is able to crosslink with MDM2 in the presence of a competing protein. This multifunctional linker, which has a terminal alkyne on the linker with a biotinylated azide, demonstrated the potential to perform pull-down assays to investigate the target selectivity of stapled peptides. The binding affinity of the stapled probe was comparable to those of previously studied p53 stapled peptides. Finally, the authors found the probe effectively photocrosslinked with MDM2 after UV irradiation, and the crosslinking was very specific for MDM2 over BSA. Currently, this methodology is limited to labeling purified proteins and known PPIs. Future directions will include MDM2 labeling and pull-down in the cell lysate or live cells.

**Fig. 9:**
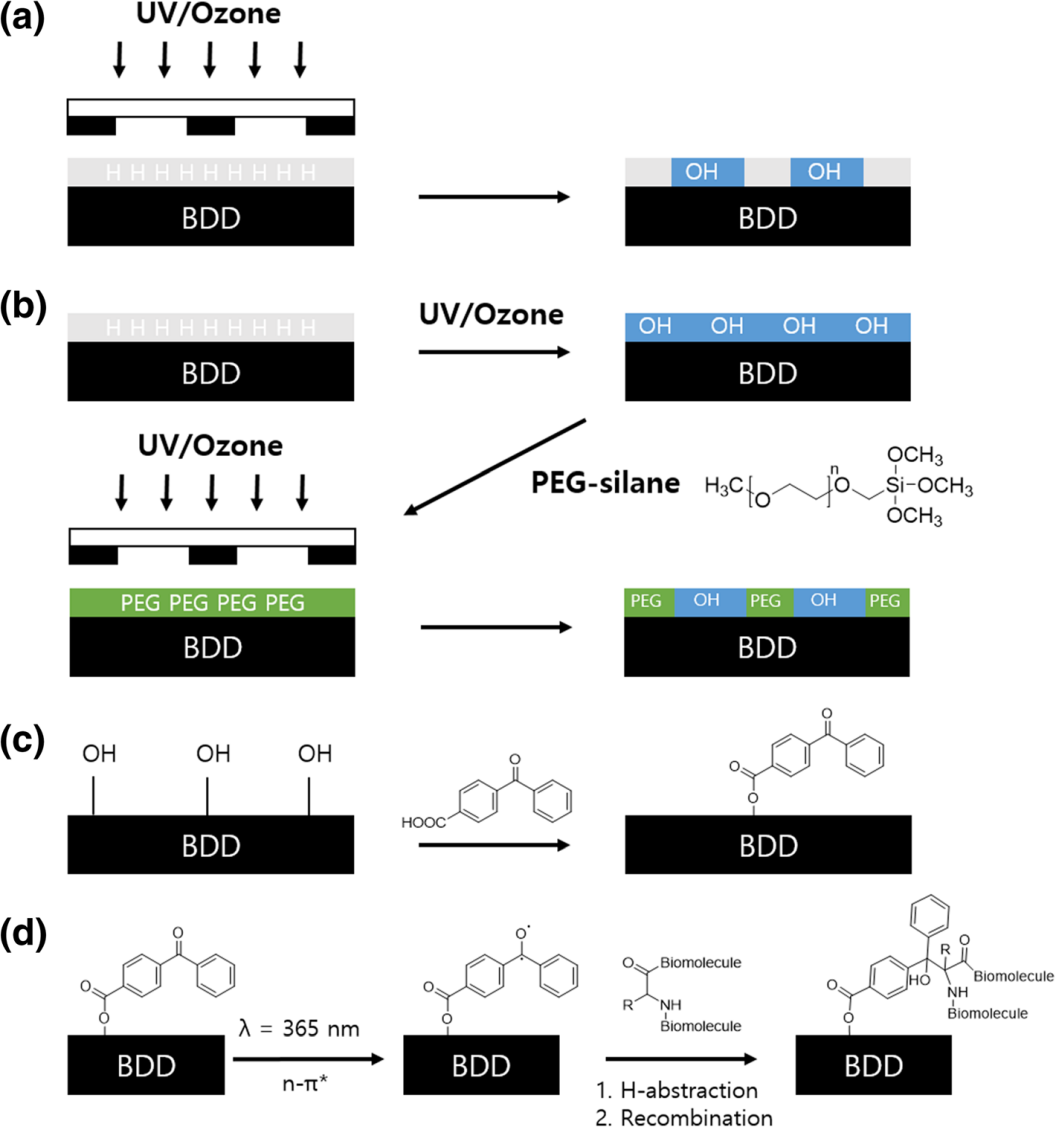
Electrochemical approach for the PAL (**a**) photolithographic oxidation of as-grown BDD, (**b**) photolitographic decomposition of mPEG-silane chains and formation of OH-BDD patterns, (**c**) esterification of OH-BDD patterns with benzophenone, and (**d**) photoimmobilization of biomolecules on benzophenone-terminated regions

Electrochemical approaches have also been used in PAL research. Sabine Szunerits et al. [52] described covalent linking of GFP and streptavidin to patterned BP-modified boron-doped diamond (BDD) electrodes (Fig. 9). Esterification was used to attach the BP moieties to the oxidized diamond surface. UV irradiation (λ = 365 nm) of the BDD surfaces in the presence of GFP or streptavidin resulted in covalent immobilization of the proteins. Nonspecific adsorption of the proteins was avoided by using poly(ethylene)glycol chains.

**Fig. 10:**
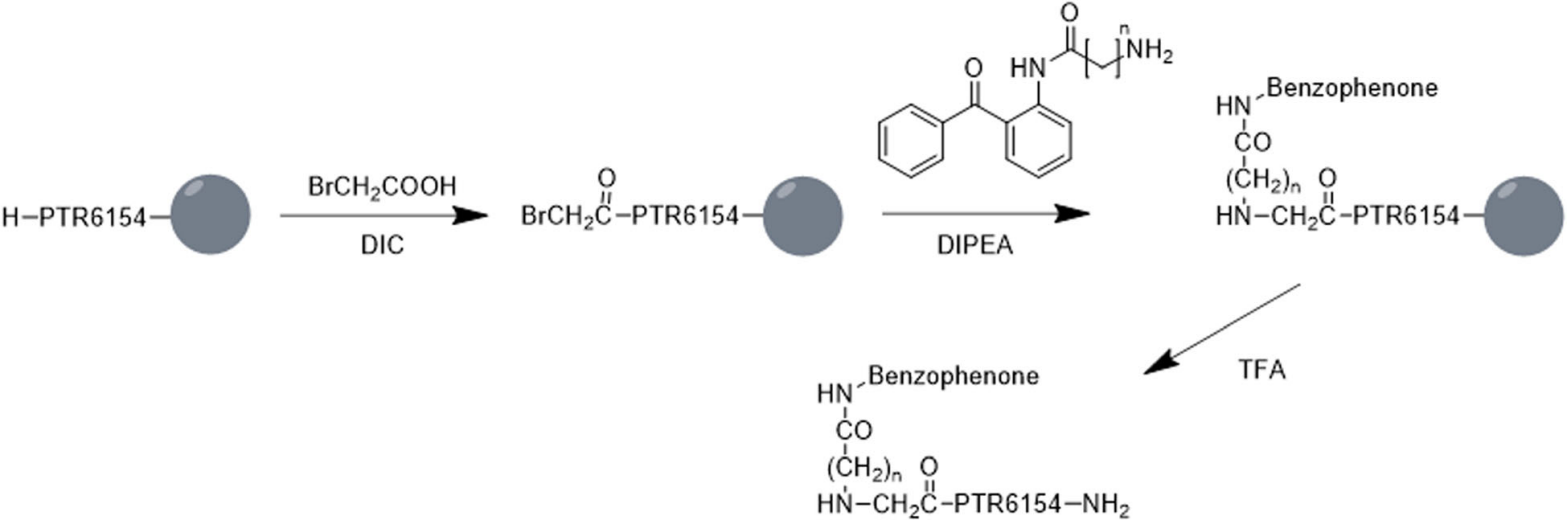
Conjugation of the BP and PTR6154 using the submonomer approach

Peptide-based probes were used by the Chaim Gilon group [53], who developed a one-pot two-step synthesis protocol for novel BP-based probes. To thoroughly investigate the binding site, linkers of various lengths were attached to the BP moiety. Solid-phase peptide synthesis (SPPS) protocols were used to incorporate these units into peptide sequences, and the method was used to prepare BPU-peptide conjugates to study the interaction between PKB/Akt and its peptide inhibitor, PTR6154 (Fig. 10). Their research demonstrated that the distance between BP and the peptide has a strong influence on the site of cross-linkage and can also affect potency. By using PAL they showed that the peptide probe could be crosslinked to an interacting protein to give the exact binding site. This method can be used to study PPIs in a variety of biological systems.

**Fig. 11:**
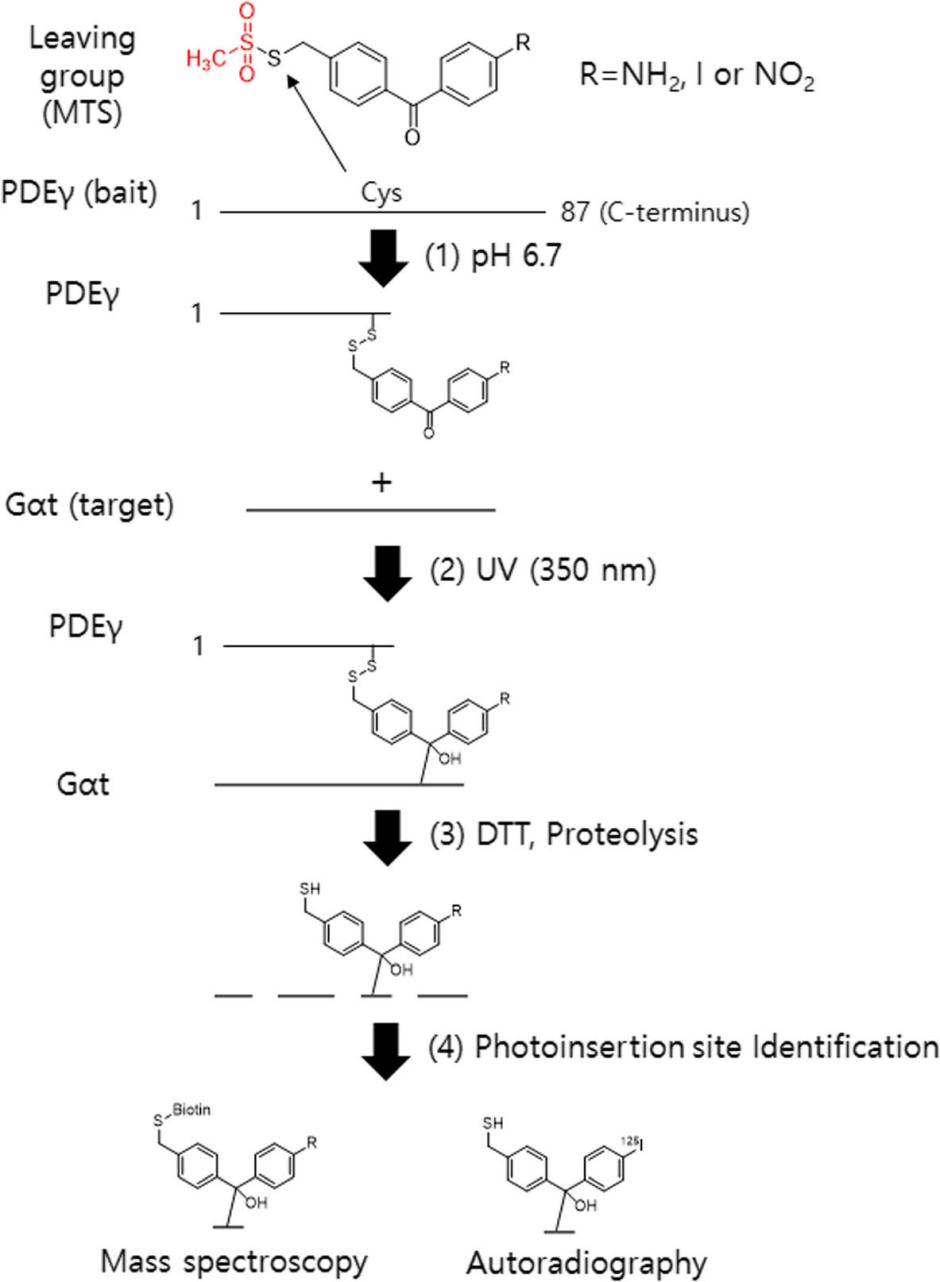
Schematic representation of PPI analysis by photo-cross-linking/label transfer using cleavable benzophenone photoprobes. PDEγ/GRt interaction is presented as a model system

Most of the studies reported for PAL are based on mass spectrometry but Lian-Wang Gua et al. have applied PAL to autoradiography. By combining the techniques, Lian-Wang Guo et al. [54] reported the development of three novel sulfhydryl-reactive BP probes with a substitution of either amino, iodo, or nitro at the para-position for direct radio-iodination. The potential use of these probes to study PPI was evaluated using the inhibitory subunit of rod cGMP phosphodiesterase (PDEγ) and the activated transducin R subunit (GRt-GTPγS) as a model system (Fig. 11). These photo-probes were stable at neutral pH and had a dithiothreitol-cleavable unit. The PDEγ covalent constructs derivatized at the C-terminal with these probes could be easily purified, and the photocrosslinking efficiency was as high as 40%. Later, the amino BP probe was radio-iodinated for autoradiography using radio-iodinated derivatives. The characteristics of radiolabeling and BP make this system more robust to study PPI by mass spectrometry when a nonradioactive label is used and by autoradiography when a radio-iodinated label is used.

**Fig. 12:**
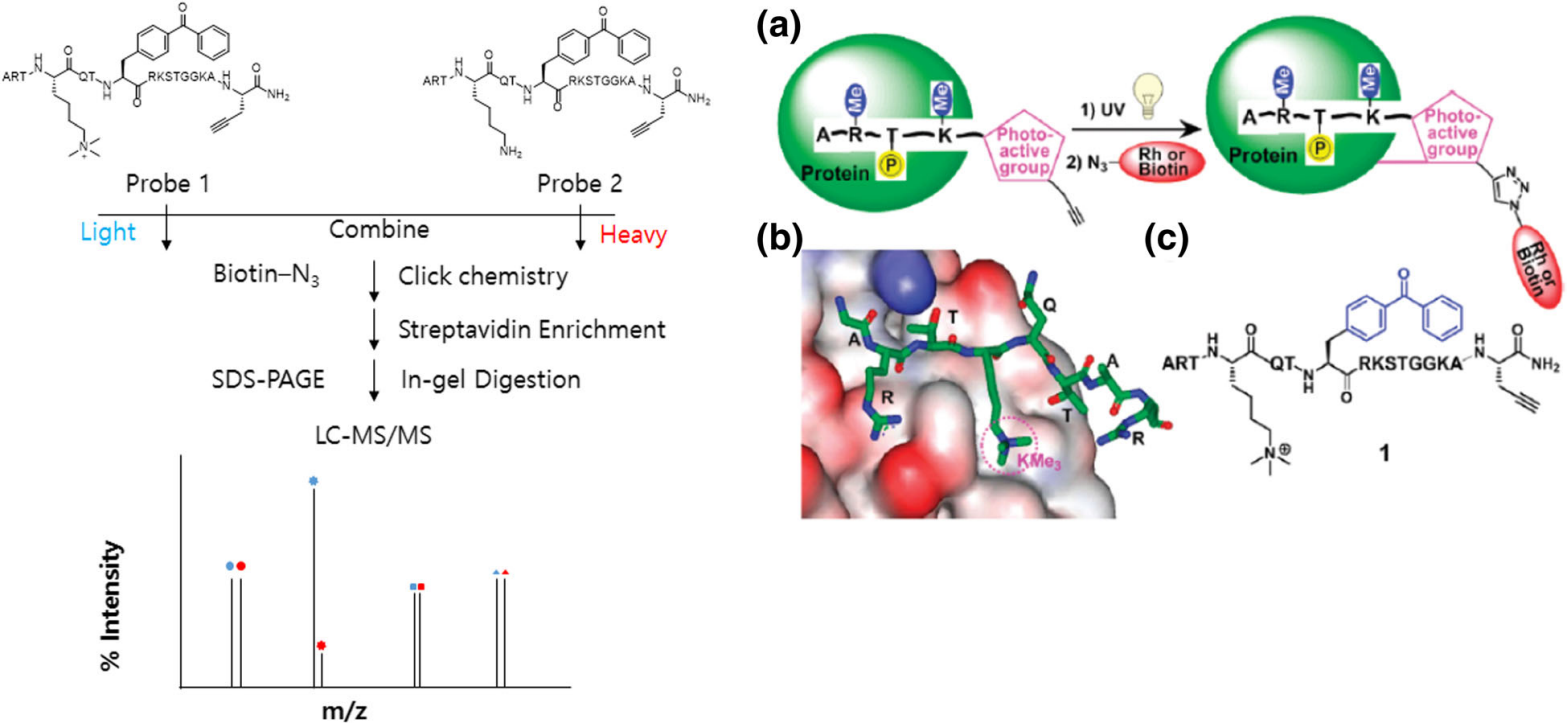
(1) Schematic representation of the CLASPI strategy to profile H3K4Me_3_ binding partners in whole-cell proteomes. **a** Strategy to capture proteins that recognize histone PTMs. **b** Structure of PHD finger of ING2 binding to a H3K4me3 peptide. **c** Chemical structure of probe 1. Figure reproduced from ref. [56] with permission from ACS publication

Quantitative chemical proteomics [55] has also been used for PAL. The Tarun M. Kapoor group used this approach to identify post-translational modification (PTM) mediated PPIs. They reported a method that combines PAL with stable isotope labeling in cell culture (SILAC)-based quantitative mass spectrometry to identify PTM-dependent PPIs. They used trimethylated lysine-4 at the histone H3 N-terminus (H_3_K_4_Me_3_), a PTM linked to actively transcribed gene promoters (Fig. 12 (1)). They identified a new protein, MORC3, along with proteins previously known to recognize this modification. This new approach of PAL-assisted and SILAC-based protein identification (CLASPI) can be used to investigate the PPIs mediated by PTMs, such as lysine methylation.

In addition to this approach, the same group [56] used the PAL with BP to profile proteins to recognize post-translationally modified histone “tails”. They used the protein structure to guide the design of the peptide probes used for irreversible covalent linkages through photocrosslinking. The photocrosslinking groups were incorporated to perturb PPIs. The PAL probe also contained an alkyne handle for “click” chemistry-mediated conjugation of reporter tags for the rapid and sensitive detection (via rhodamine) or affinity enrichment (via biotin) of labeled proteins. To evaluate their approach, the authors prepared an affinity handle by trimethylation of lysine-4 of histone H3 (H3K4me3), an evolutionarily conserved and well-characterized PTM at the N-terminal region of the histone. In combination with their approach using mass spectrometry, they profiled proteins for recognition of histone modifications. This approach could be used to identify the “readers” of combinatorial modifications linked to specific biological states (e.g., lysine-9 trimethyl and phosphorylated ser-10 of histone-3 during mitosis). Additionally, this methodology can be extended to identify the proteins that recognize other PTMs, particularly when these modifications are dynamic or are mediators of weak interactions (Fig. 12a-c).

**Fig. 13:**
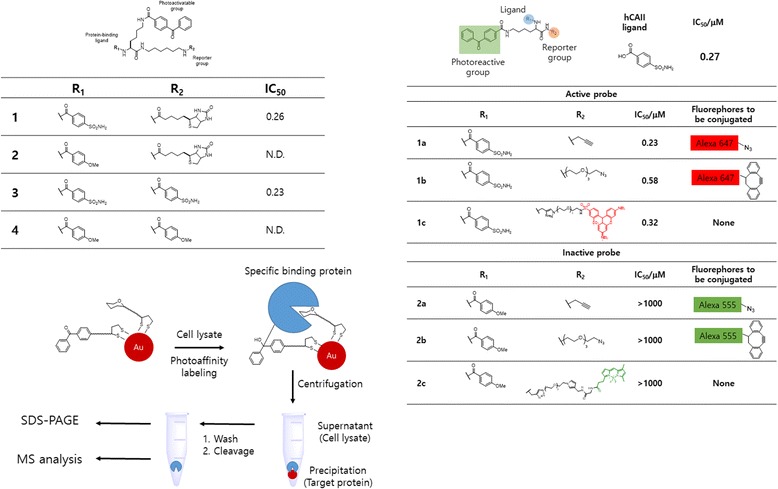
**a** Structures of the active PAL probe 1 and the inactive probes 2–4 and their inhibitory activity against hCAII (mM) **b** Active/inactive PAL probes, their hCAII inhibitory potencies (IC 50), and fluorophore-conjugated click reagents for the reactions. **c** A efficient photo-affinity labeling approach toward identification of carbohydrate-binding proteins by using AuNP-based multivalent carbohydrate probes

The Kaori Sakurai group [57] extensively used the PAL of carbohydrate-binding proteins. They developed an active/inactive dual probe approach that can control the selectivity of PAL reactions to detect specific small-molecule-binding proteins. This approach can also be applied to lower-level binding proteins in a cell lysate. In addition, they found that a simple inactive analog representing the scaffold moiety of the PAL probe can improve the labeling selectivity. To complete their approach, they developed probes 1–4 (Fig. 13a). To identify the binding protein of benzenesulfonamide by PAL, they designed trifunctional probe 1 based on an l-lysine scaffold that contains a benzenesulfonamide moiety as a protein-binding ligand, BP as a photoactivatable group, and biotin as a reporter group, which enables detection of the protein–covalent adduct. Compound 3, which contains the ligand group but lacks biotin, was used as a positive control. Compounds 2 and 4 represented inactive analogs. A similar approach was used by the same group [58], using an active/inactive dual-PAL system for selective crosslinking and straightforward detection of small-molecule-binding proteins. They designed a novel PAL reaction in which nonspecific proteins were scavenged by an inactive probe and co-reacted with a conventional PAL probe. This new method can be used to selectively detect specific binding proteins at levels as low as 0.1% (w/w) in the cell lysate using either 1D or 2D electrophoresis (Fig. 13b). In addition to the probes, Kaori Sakurai et al. [59] developed gold nanoparticle-based multivalent carbohydrate probes for selective PAL of carbohydrate-binding proteins. The probes were assembled using AuNPs as scaffolds, a carbohydrate ligand and a photoreactive group in a modular fashion (Fig. 13c). The novel AuNP-based probes served dual functions of easing PAL and directly enriching the crosslinked proteins by centrifugation. They demonstrated that their ability to enhance the affinity and selective PAL could be easily enhanced by removing nonspecific proteins, which enabled the isolation of a low-affinity carbohydrate-binding protein in the cell lysate. According to them, this was the first example of a streamlined PAL approach where crosslinking, enrichment and isolation of binding proteins were performed using a single probe.

**Fig. 14:**
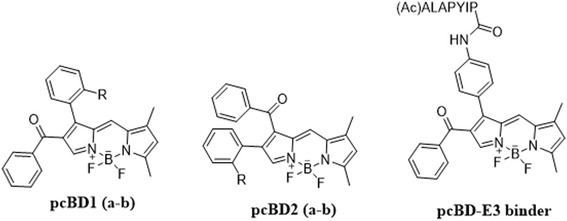
Structures of photo-crosslinking BODIPY (pcBD) probes

In the same regard, we developed the first rational design of a photocrosslinking BODIPY fluorophore (pcBD) [60] (Fig. 14) and its biological application for biomolecule labeling. As a photosensitizing functional motif, an aryl ketone group was incorporated into the BODIPY fluorophore, and a series of proteins were labeled with pcBD compounds upon UV irradiation. Compared with the conventional dual-tagging approach, pcBD tagging provides a major advantage in terms of a small versatile tag. As a proof-of-principle, we synthesized amino-functionalized pcBD, which was covalently attached to the ubiquitin ligase binding peptide (ALAPYIP). Upon UV irradiation, we could visualize the substrates in the total lysate. However, its application is not limited to a specific enzyme. Any enzyme-ligand could be attached to pcBD to visualize substrates. Such efforts could contribute significantly to enhancing PPIs in complex biological systems. Currently, we are developing an approach by changing the substrate affinity handle.

## Diazirine-based probes

The small size of DA makes this PL special because it can be incorporated very close to the active site. The ultimate effect is increased photocrosslinking efficiency compared with other PLs. Different types of DA-based probes have been developed using different approaches to PAL. These probes are reviewed below.

**Fig. 15:**
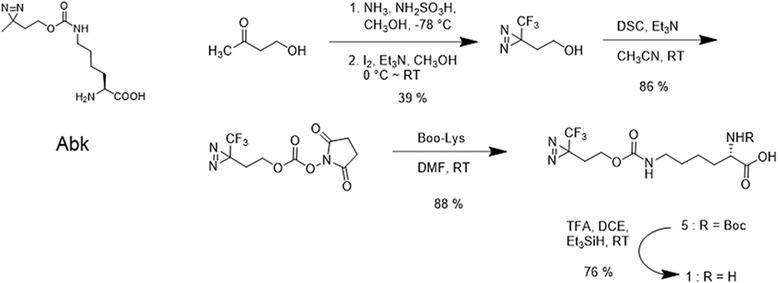
The chemical structure of 3′-azibutyl-N-carbamoyl-lysine (AbK) and Synthesis of the diazirine-modified lysine

Like BP-based genetically encoded amino acid probes, DA-based genetically encoded amino acid probes have also been developed. Peter G. Schultz et al. [61] studied PPI with a genetically encoded photocrosslinking amino acid. They engineered wt-mbPylRS/tRNA^Pyl^ to genetically encode the aliphatic photocrosslinking amino acid AbK (Fig. 15) with high efficiency in both E. coli and mammalian cells. The small size and flexible nature of AbK may cause less structural perturbation than other photocrosslinking amino acids when incorporated into proteins. Additionally, they believe the improved efficiency of AbK incorporation could be useful when the target POI is difficult to express. In other studies, Alexander Deiters et al. [62] used genetically encoded aliphatic DA for protein photocrosslinking and PAL. They reported a novel aliphatic DA amino acid and its genetically encoded, site-specific incorporation into proteins in bacterial and mammalian cells (Fig. 15). Additionally, they demonstrated efficient PAL of a test protein in vitro and in vivo. The authors also demonstrated that the pyrrolysyl-tRNA synthetase/tRNA CUA pair could be used to introduce the lysine-based DA amino acid into proteins in E. coli and mammalian cells in response to the amber codon, TAG, with good yield.

**Fig. 16:**
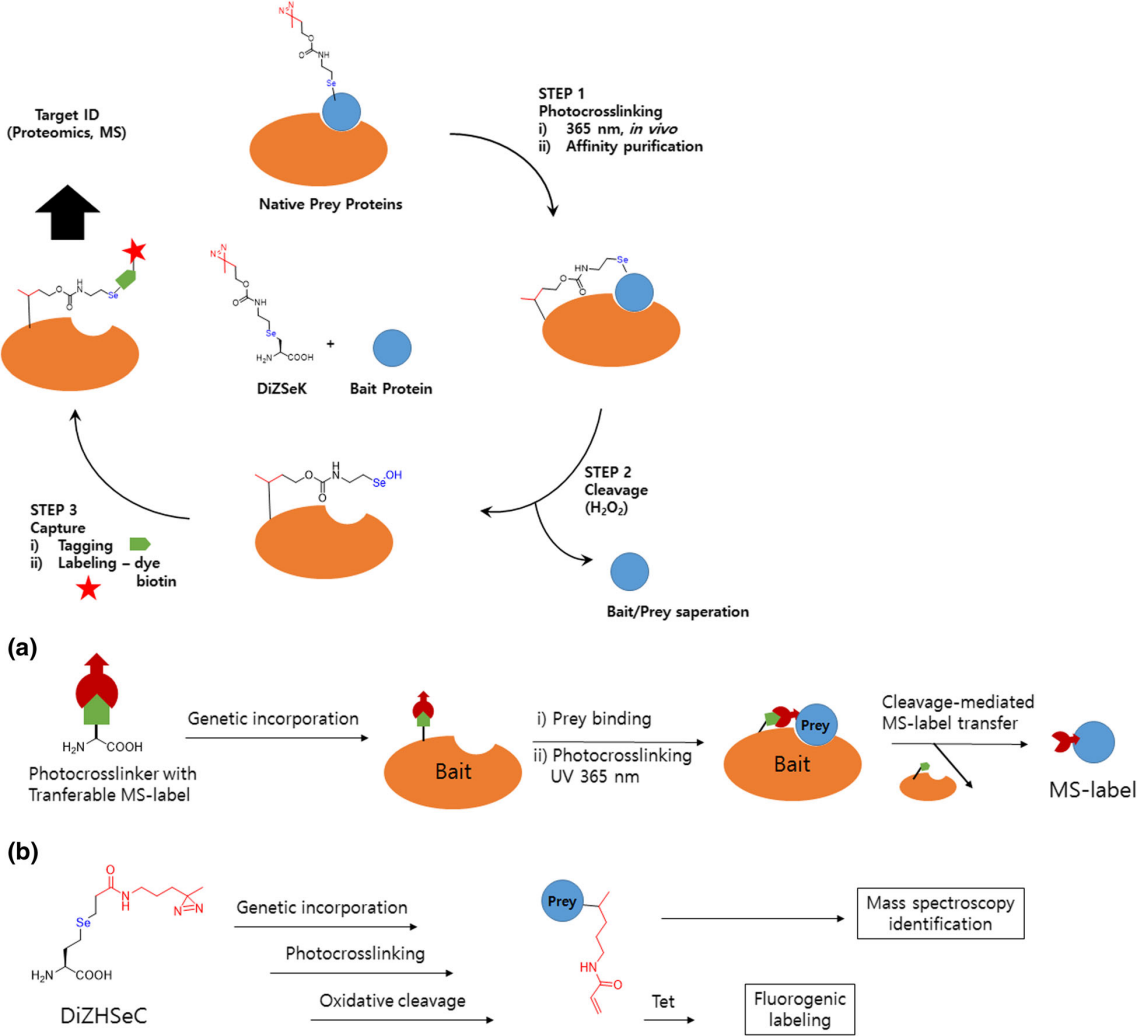
(*Top*) A General Procedure for Protein Photo-Cross-Linking Using a Cleavable Photo-Cross-Linker. (**a**) In situ generation of MS-label on prey proteins by using a genetically encoded cleavable photocrosslinker. (**b**) Chemical design of the photocrosslinker (DiZHSeC) with transferable MS-label

Another genetically encoded amino acid was developed by Peng R Chen et al. [63] they developed a genetically encoded, selenium-based cleavable photocrosslinker probe for the discrimination of bait and prey proteins upon PAL. They employed pyrrolysine-based genetic code to encode a Se-containing cleavable protein photocrosslinker and developed a cleavage and in situ capture of interaction CAPP strategy (Fig. 16 left). This cleavable photo-affinity amino acid can covalently trap prey proteins under living conditions and allows for the subsequent separation of bait and prey proteins via H_2_O_2_-mediated oxidative cleavage. The released prey proteins carry an in situ-generated selenenic acid, which will be further captured by (i) tagging with an alkyne-bearing DMA molecule and (ii) labeling with an azide-containing fluorophore or biotin probe. This cleavage and capture after protein PAL method enable the capture of prey proteins that are readily accessible by 2D gel-based proteomics and mass spectrometry. The authors studied this concept by profiling the in vivo binding proteins of an E. coli acid chaperone HdeA under acid stress. In other studies, the same group [64] developed a new method for PPI studies in which a genetically encoded photo-affinity unnatural amino acid was introduced to a mass spectrometry-identifiable label (MS-label) (Fig. 16 Right) to capture prey proteins after photocrosslinking and prey-bait separation. This strategy, named IMAPP (in situ cleavage and MS-label transfer after protein photocrosslinking), could be directly used to identify photo-captured substrate peptides, which are difficult to uncover using conventional genetically encoded photocrosslinkers. Considering this advantage of the MS-label, the IMAPP strategy significantly enhances the confidence in identifying PPIs and enables simultaneous mapping of the binding interface under living conditions.

**Fig. 17:**
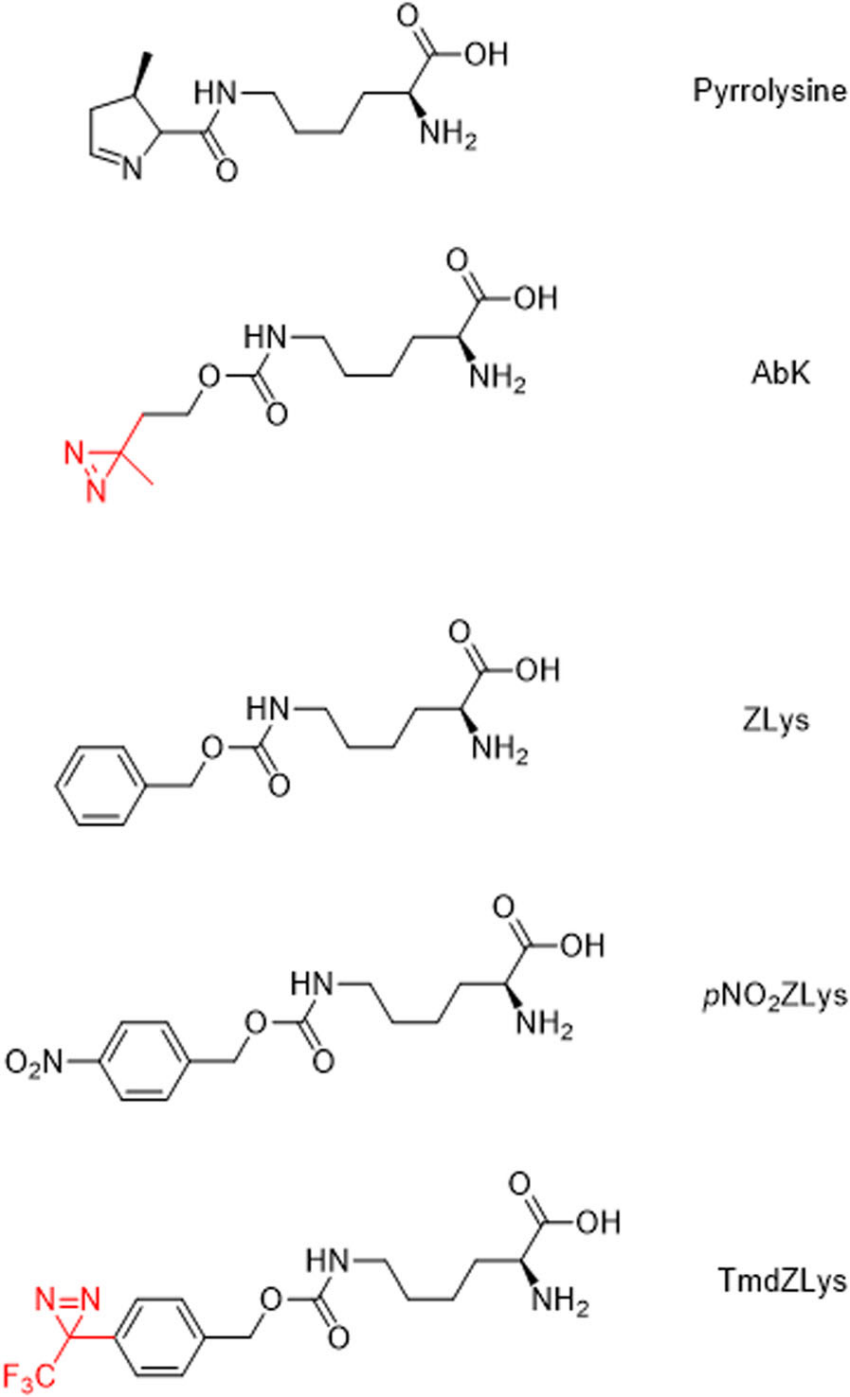
Chemical structures of pyrrolysine (1), AbK (2), ZLys (3), pNO_2_ZLys (4), and TmdZLys (5) with DA as PL

In another study, Shigeyuki Yokoyama et al. [65] developed a derivative of N^ε^-benzyloxycarbonyl-L-lysine with a photoreactive diazirinyl group, N^ε^-[((4-(3-(trifluoromethyl)-3H-diazirin-3-yl)-benzyl)oxy)carbonyl]-L-lysine (Fig. 17), which was further site-specifically incorporated into target proteins in mammalian cells. This genetically encoded photocrosslinker, which can react with residues as distant as approximately 15 Å, also reacts with those in closer proximity, enabling “wide-range” photocrosslinking of proteins. The probes presented here are the first probes for long-range protein crosslinking with a lysine derivative. The probe TmdZLys, with the longest linker between the C_α_ and the reactive center, is far reaching and can react with residues nearby. These properties of TmdZLys increase the photocrosslinking efficiency, which could help to identify the binding interfaces between proteins.

**Fig. 18:**
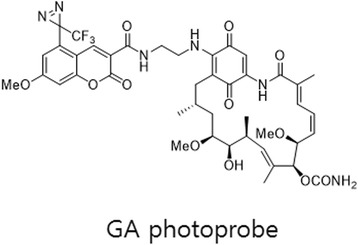
Chemical structure of GA photoprobe

In addition to the use of DA as PL, researchers have demonstrated the use of coumarin as a fluorophore along with DA to study PPIs. The Yasumaru Hatanaka group [66] developed a coumarin-based DA probe that shows a dramatic increase in fluorescent intensity upon photocrosslinking with the POI. A DA group quenched the FL intensity probe, but it was recovered upon UV irradiation at 365 nm. As a proof of concept, the authors installed their coumarin-DA adduct at C-17 of geldanamycin (GA) (Fig. 18), which is a potent inhibitor of heat shock protein 90 (Hsp90). This photoactivatable GA probe, upon UV irradiation at 365 nm, showed specific labeling and visualization of Hsp90 as a binding protein. Therefore, by using this methodology, one can install a fluorophore at the interacting interface, which would be useful for identifying a ligand-binding domain within a target protein.

**Fig. 19:**
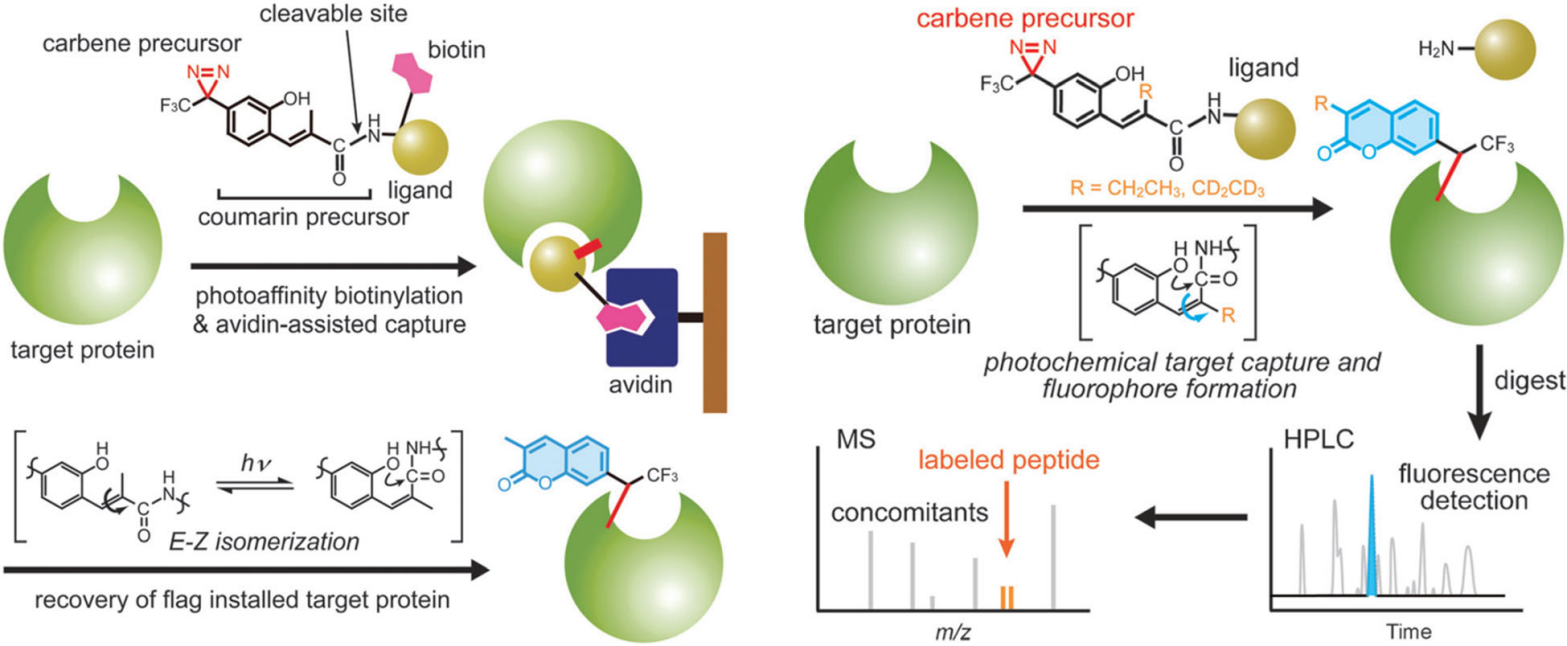
(*left*) Tag-switching strategy for the identification of target proteins by double photoreactions of a multifunctional cross-linker. Figure reproduced from ref. [67] with permission from RCS publication. (*Right*) A new strategy for target identification using PAL with IsoFT simplifies the identification of the target peak in both HPLC and MS analyses. Figure reproduced from ref. [68] with permission from Wiley-WCH publication

In continuation of this study, the same group [67] developed a coumarin-based photoswitchable fluorescent flagging approach to identify PAL peptides in the target protein (Fig. 19 left). This method simplified the complex identification process for labeled sites. The amount of protein required for analysis is in the 10 micrograms range. The other key feature of this approach is the identification of multiple peptide components of a binding domain that were not characterized by conventional photolabeling approaches. This label-switching strategy could be used to identify target proteins in low abundance, which is a major challenge in techniques such as shotgun proteomics. Takenori Tomohiro et al. [68] also used coumarin-based probes. They described an isotope-coded fluorogenic crosslinker for high-performance target identification based PAL. In PAL, a high-performance chemical tag, an isotope-coded fluorescent tag (IsoFT), is attached to the interacting sites by irradiation to rapidly identify target proteins (Fig. 19 Right). The authors developed a stable isotope-based method using their fluorophore-tagging technique, which uses an isotope-coded fluorescent tag (IsoFT) as a fully functionalized crosslinker for rapid identification of labeled peptides without requiring highly purified targets.

**Fig. 20:**
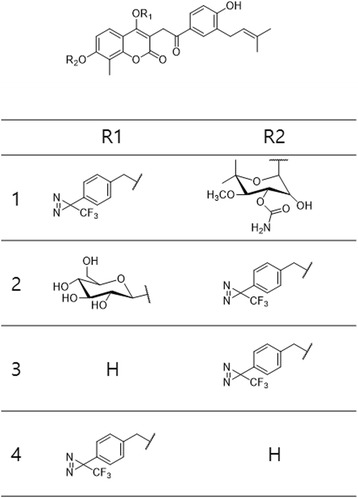
Chemical structures of coumarin based PLs

The Min Yang group [69] also used coumarin-based probes to synthesize and characterize the glucosyl-novobiocin-based DA PAL reagents for the Hsp90 C-terminal domain. They synthesized five PALs (Fig. 20) from novobiocin in six consecutive steps using phase-transfer catalytic glycosylation. 2D-NMR spectroscopy and MS were used to analyze the structures and bonding linkages of these compounds. This glucosyl-novobiocin alteration strategy provides a valuable method for further development of enhanced glucosyl novobiocin mimetics. Preliminary data show binding between Hsp90CTD and compound 1, and MS analysis data identified the exact peptide bound to the PAL. This method could be used as an effective synthetic route for multifunctional compounds and as a simple chemical biology tool to probe the unknown protein binding pocket SAR.

**Fig. 21:**
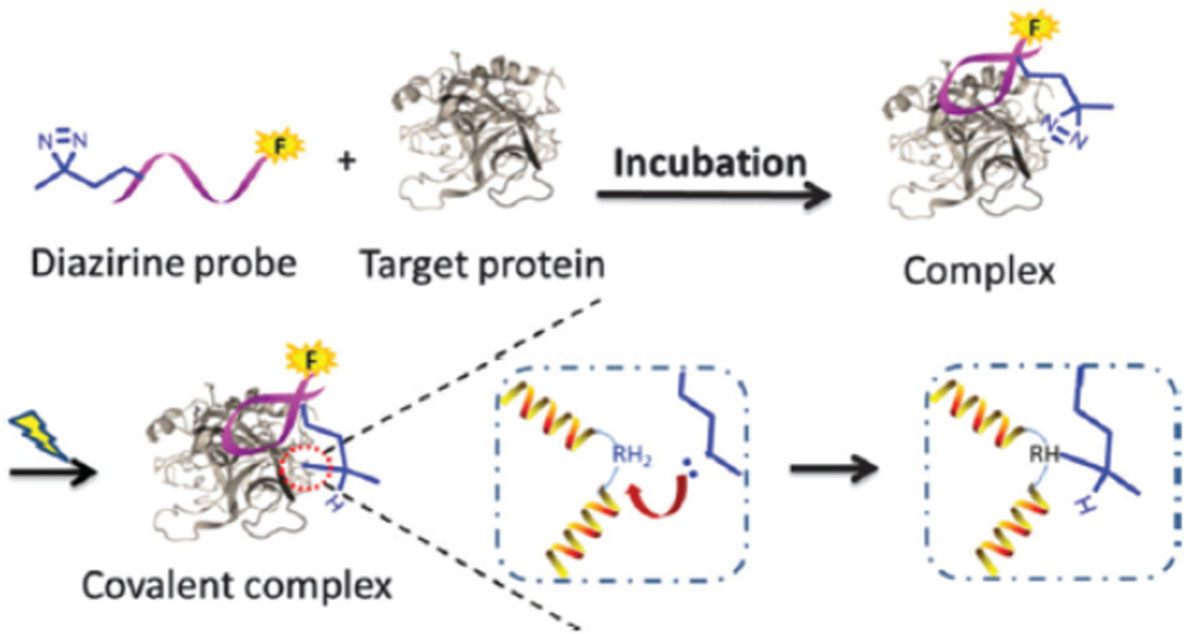
Photo-initiated efficient covalent coupling of diazirine modified aptamer probe with its target protein for biomarker discovery. Figure reproduced from ref. [70] with permission from RSC publication

Another approach is the use of a DA-based aptamer for PPI studies. Chaoyong James Yang et al. [70] demonstrated a photoactivatable labeling reagent, DA phosphoramidite, for easy and flexible site-specific labeling of DNA ligands with the DA moiety. This DA phosphoramidite (diazidite) was chemically synthesized and used for easy and flexible site-specific labeling of a DNA sequence with an automated DNA synthesizer (Fig. 21). The resulting DA-labelled aptamer was used to form a covalent bond between the aptamer and the target upon 365 nm irradiation. As a proof-of-concept, the authors selected two known aptamer targets, streptavidin (SA) and thrombin (TMB), to verify the feasibility of the photocrosslinking capability of DA-labelled aptamers with target proteins. Additionally, they compared the photocrosslinking efficiency of their probe with that of the widely used I-dU probe. As a result of photolysis, they found the DA-modified streptavidin and thrombin aptamers have high efficiency and specificity for photocrosslinking with their corresponding target proteins. The diazidite probe can thus be used for biomarker discovery by PAL-based covalent labeling of biomarkers with aptamers generated from cell-SELEX.

**Fig. 22:**
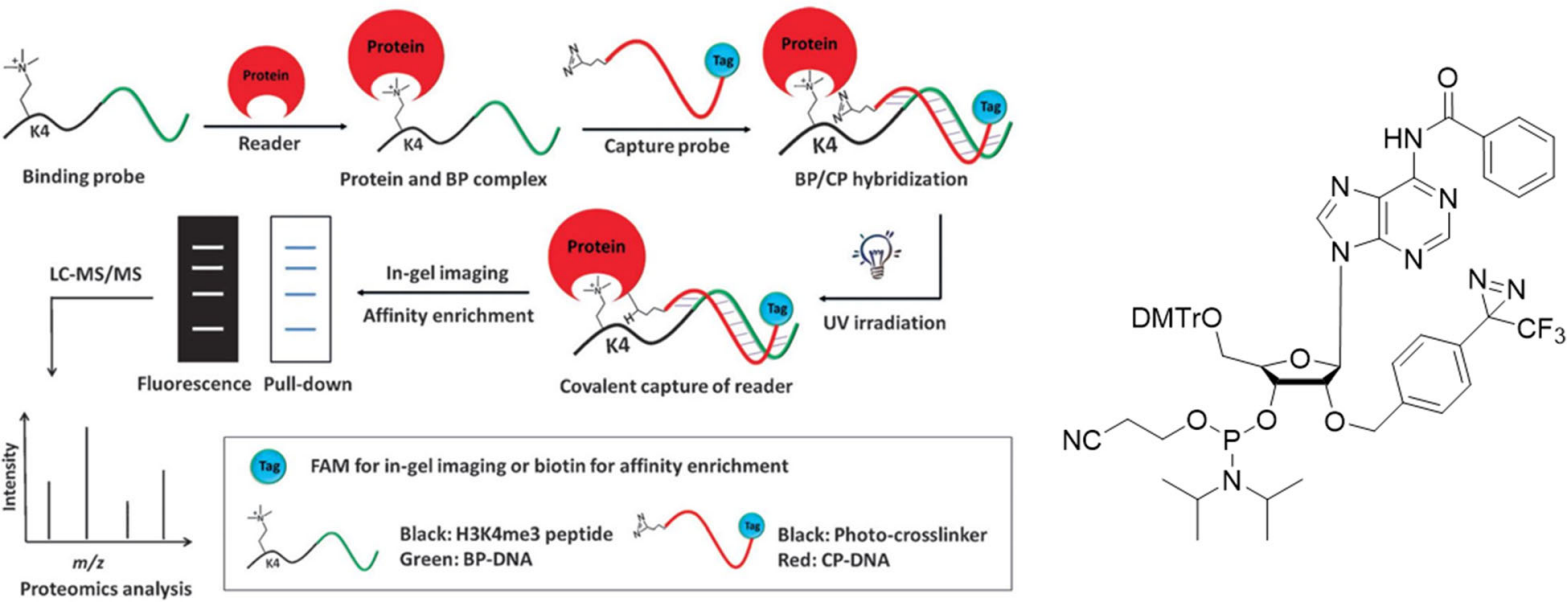
(*Left*) preparation and application of the HPTM dual probe, based on DNA-templated chemistry and photo-crosslinking, for the identification of HPTM reader proteins. Figure reproduced from ref. [71] with permission from Wiley-WCH publication. (*Right*) structure of a ^D^A phosphoramidite unit

DNA-templated peptide probes have been developed by the Kai Zhang group [71]. They developed a DNA-templated peptide probe for PAL and enrichment of the histone modification reader proteins (Fig. 22 left). They combined DNA-templated technology with a DA as the photocrosslinking agent to design an HPTM dual probe as a novel HPTM peptide-based PAL approach for the identification of histone readers. This dual probe provides flexibility for HPTM affinity through DNA-templated chemistry, and the DA provides PAL for covalent interactions with even low-affinity reader proteins by photocrosslinking without affecting the binding efficiency between HPTMs and the readers. Akio Kobori et al. [72] developed novel photoresponsive oligodeoxyribonucleotides with a 2′-O-DA-conjugated adenosine for DNA interstrand crosslinking (Fig. 22 Right). Photocrosslinking studies of ^D^A- containing oligodeoxyribonucleotides with complementary oligo-DNAs and oligo-RNAs revealed that oligodeoxyribonucleotides reacted exclusively with DNAs. Photocrosslinking studies revealed that the DA-containing oligodeoxyribonucleotides selectively crosslinked with the oligo-DNAs (and not with the oligo-RNAs), with only 5 min of UV irradiation required for near completion of the photocrosslinking reactions.

**Fig. 23:**
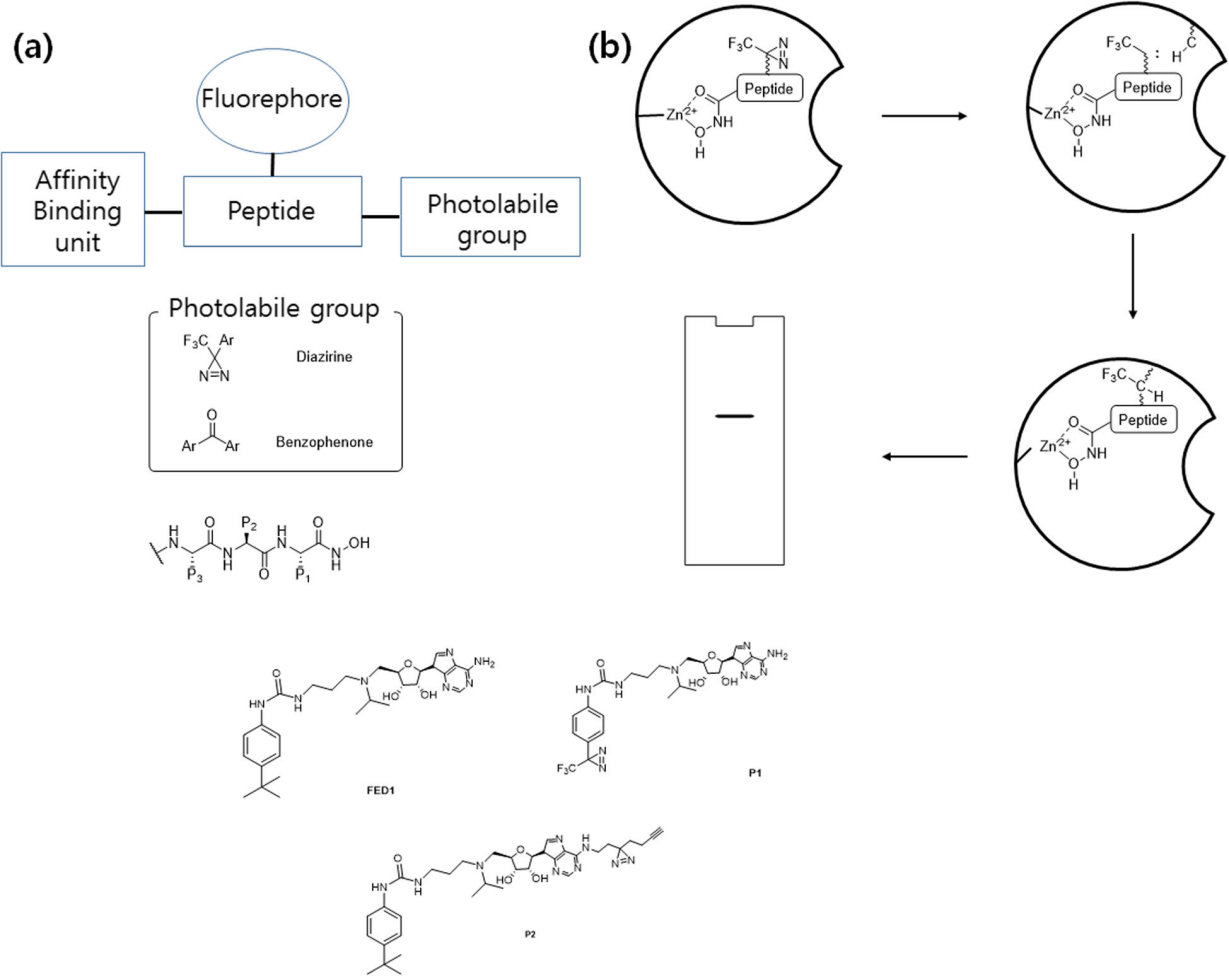
([*Top* (**a**, **b**)]) Schematic representation of probes for affinity-based proteomic profiling; **b** schematic representation of affinity-based profiling of metalloproteases (*Bottom*) Structure of FED 1 and the probes P1 and P2

An affinity-based labeling approach was proposed by the Yao group [73]. They described an affinity-based labeling approach for enzyme profiling that does not contain covalently bound substrate intermediates. Their probe design consists of the peptidyl hydroxamate zinc-binding group (ZBG) of the metalloproteases, a fluorescent reporter tag, and a DA group as photocrosslinker (Fig. 23 Top (a, b)). Photolysis of the DA in the probe effectively generates a covalent adduct of the probe and the target enzyme, making the enzyme distinguishable from unlabeled proteins upon separation on a SDS-PAGE gel. They chose DA as the photocrosslinker unit over BP because the DA-based probes were able to label a small amount of the model metalloprotease from crude yeast extract selectively with high sensitivity and low background labeling. The same group [74] recently designed and synthesized a PAL reagent and “clickable” affinity-based probes (AfBPs) with cell-permeability and structural mimics of FED1 (Fig. 23 bottom). FED1 is a small-molecule inhibitor of DOT1L [protein methyltransferase that methylates histone H3 on lysine 79 (H3K79) and is a promising drug target against cancers]. FED1 is also a potential anticancer agent and can be used to investigate the biological roles of DOT1L in human diseases. For the first time, the authors showed that by using their newly designed probes, they could conduct the cell-based proteome profiling followed by quantitative LC-MS/MS experiments to identify potential cellular off-targets of FED1.

**Fig. 24:**
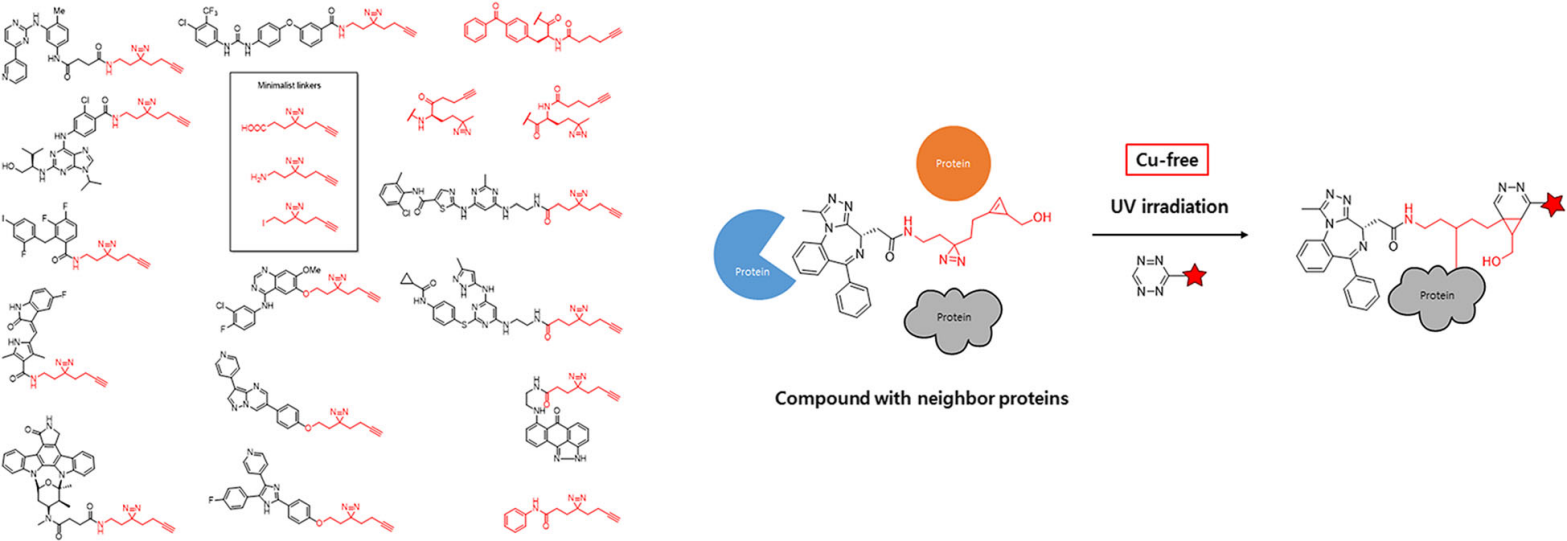
(*left*) Chemical structures of the 3 “minimalist” linkers and 12 corresponding kinase probes (*Right*) second-generation approach reported in the current work, with cyclopropenes as chemically tractable tags suitable for copper-free bio-orthogonal chemistry

The same group [75] developed three minimalist terminal alkyne-containing DA photocrosslinkers. To validate the application of their probes in chemical proteomics, they synthesized 12 linker-modified kinase inhibitors (Fig. 24 left), which were used for cell-based proteome profiling of potential cellular kinase targets. The probes could be applied under various conditions in different biological systems. Various potential off-targets of these probes were identified, some of which were confirmed by Western blotting. In 2014, the same group [76] used the “minimalist” cyclopropene-containing photocrosslinkers suitable for live-cell imaging and affinity-based protein labeling. They developed novel minimalist linkers containing both an alkyl DA and a cyclopropene (Fig. 24 Right) and showed that chemical probes made from such linkers could be used for in situ imaging and covalent labeling of endogenous BRD-4 protein via a rapid, copper-free, tetrazine-cyclopropene ligation reaction. The key feature of these cyclopropenes is their unique C-1 linkage to a BRD-4-targeting moiety, enabling highly tunable reactivity, solubility, relative stability, and synthetic accessibility. BD-2, which is a linker-modified analog of (+)-JQ1 (PPI inhibitor of BRD-4), was subsequently used in a cell-based proteome profiling experiment for large-scale identification of potential off-targets of (+)-JQ1. Several newly identified targets were also confirmed by preliminary validation experiments.

**Fig. 25:**
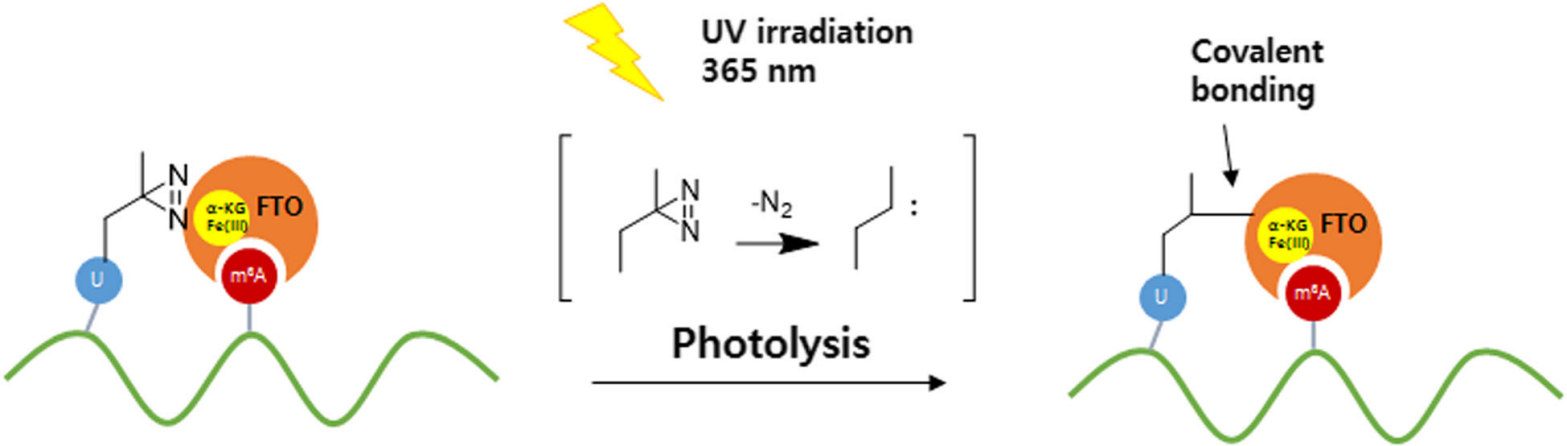
FTO recognition mechanism of m^6^A and the design of diazirine photocrosslinking between the m^6^A-containing RNA and FTO

Akimitsu Okamoto et al. [77] attached a DA photo-affinity group to m^6^A recognition mediated by the fat mass and obesity associated (FTO) demethylase. DA-induced PAL dramatically enriched the activated FTO-based m^6^A recognition complexes (Fig. 25), which formed within the first 10 min of the reaction. The PAL data indicate that cofactor Fe(II), accompanied by α-KG, determines the ability of FTO to discriminate between m^6^A and A. The α-KG co-substrate and the Fe (II) cofactor formed activated FTO complexes that contributed to capturing dioxygen and recognizing m^6^A. In addition, the reaction mechanism of FTO reveals that ssRNA binds to FTO first, independent of the m^6^A substrate, and then m^6^A recognition occurs by oxidative demethylation. This method proved that the enrichment of the photocrosslinked ssRNA-FTO complex is independent of the m^6^A or A substrate. Finally, DA PAL proved to be useful to capture activated FTO-mediated oxidative demethylation.

**Fig. 26:**
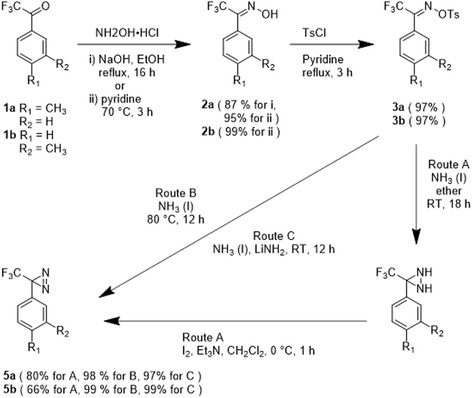
Synthesis of 3-(m-or p-tolyl)-3-(trifluorometh-yl)-3H-diazirines

Photoreactive saccharin derivatives were developed by the Makoto Hashimoto group [78]. They designed and synthesized photoreactive saccharin derivatives that contain a (trifluoromethyl)diazirinyl moiety at the 5- or 6-position (Fig. 26) for use as functional analysis tools for PAL to elucidate the sweet and bitter taste mechanisms. The data showed that the preparation of the diazirinyl-saccharin derivatives was effective and that these photoreactive compounds had sufficient affinity for the sweet and bitter taste receptors to elucidate the binding sites of their ligands. This strategy could be used to understand the underlying molecular mechanisms of gustatory receptors.

**Fig. 27:**
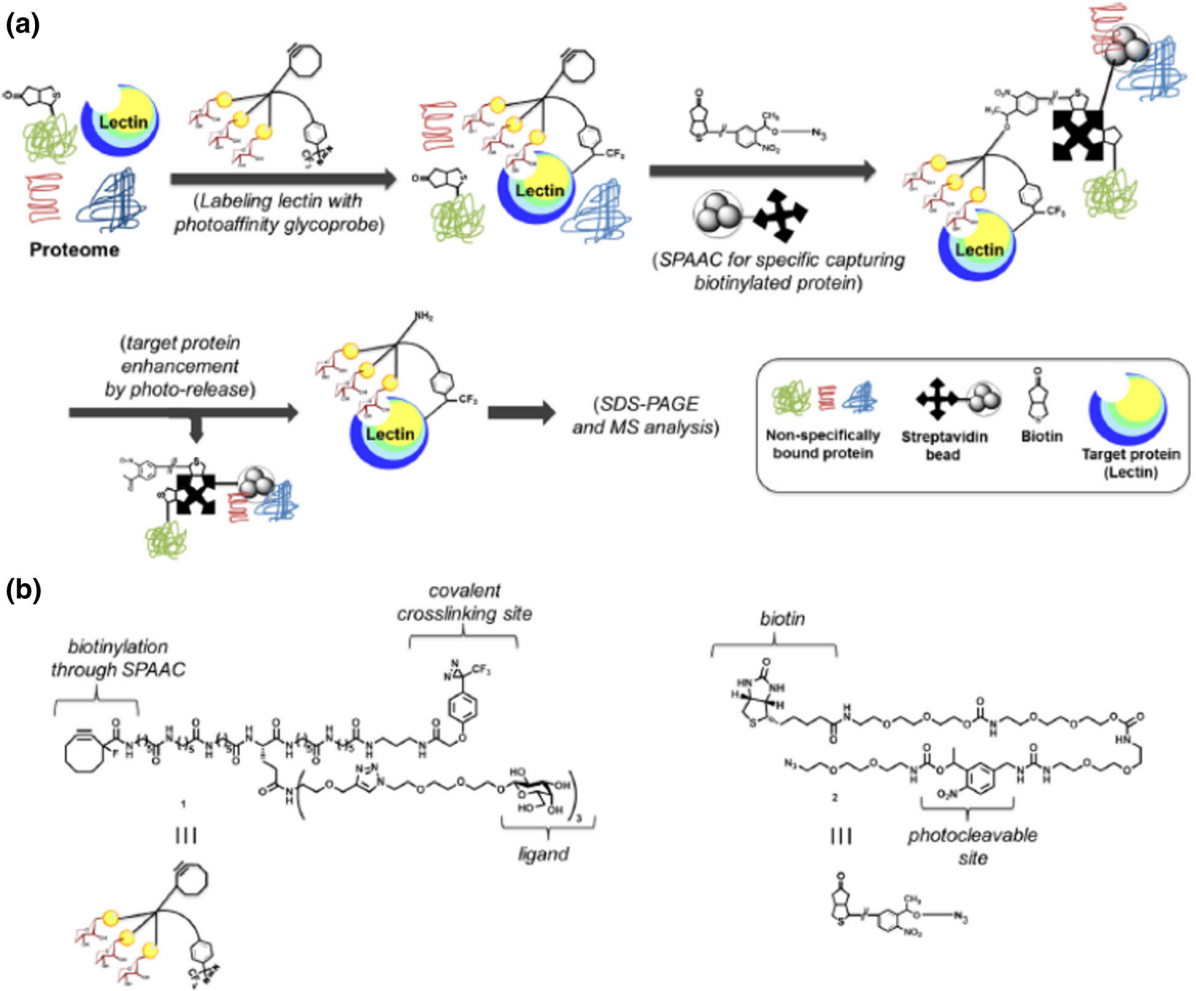
(**a**) Schematic representation of the procedure for PAL of lectins with carbohydrate photoprobe and isolation of photo-crosslinked proteins via tandem application of SPAAC, biotin-streptavidin enrichment and a photo-release step. (**b**) Design of the multivalent photo-affinity glycoprobe (1) and the photo-cleavable biotin affinity tag (2). Figure reproduced from ref. [79] with permission from ScienceDirect publication

DA-based probes have also been reported to study carbohydrate-protein interactions. In this regard, Chun-Cheng Lin et al. [79] developed photocrosslinking glyco-probes (Fig. 27) for the covalent capture of labile protein complexes to study carbohydrate-mediated interactions. They hypothesized that light activation could be exploited to release labeled proteins in a dual photo-affinity probe-based strategy. To investigate their strategy, a trifunctional, galactose-based, multivalent glyco-probe was developed for affinity labeling of carbohydrate-binding proteins. The resulting covalent protein-probe adduct was attached to a photocleavable biotin affinity tag, and the photolabile linker enabled the release of the labeled proteins. The ability of the dual probe for labeling and facile cleavage of the target protein complexes from solid surfaces eliminates some of the common drawbacks of traditional affinity-based purification methods. As a proof of principle, they designed probes based on (i) a trivalent Gal unit for affinity binding to the target lectin, (ii) a DA photocrosslinking agent, and (iii) a cyclooctyne functionality for well-established strain-promoted [3 + 2]-azide-alkyne cycloaddition (SPAAC).

**Fig. 28:**
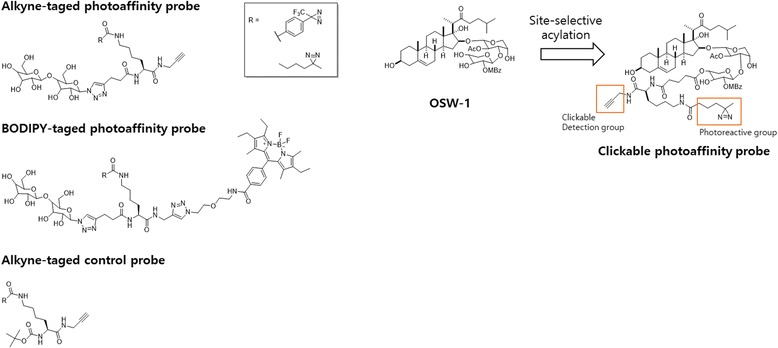
(*Left*) Structures of lactose-based photo-affinity probes and control probes bearing TPD or alkyl diazirine groups. (*Right*) Synthetic plan for synthesizing clickable photo-affinity probe1 by site-selective acylation of OSW-1. MBz = 4-methoxybenzoyl

In their continuous effort to develop PAL probes for carbohydrate-binding protein-based PAL, the authors developed DA-based probes. The Kaori Sakurai group [80] conducted a comparative study of the reactivity of DA-based PAL probes for carbohydrate-binding proteins. They synthesized a set of lactose-based photo-affinity probes with both alkyl DA and trifluoromethylphenyl DA (TPD) to compare their efficiency for the photocrosslinking of a carbohydrate-binding protein (Fig. 28 left). The probes include an alkyne tag to label an azide-conjugated fluorescent tag via Cu (I)-catalyzed azide-alkyne cycloaddition (CuAAC) following PAL. Probes 3 and 4 were synthesized with a boron-dipyrromethene (BODIPY) group so that the probe-crosslinked products could be analyzed by in-gel fluorescence imaging. For the control experiment, probes 5 and 6, which lacked a ligand moiety, were also prepared. The crosslinking efficiency data showed that the TPD probe efficiency was higher than that of the alkyl DA probes when reacted with a single binding protein. However, an alkyl DA probe with a small alkyne tag was a more selective PAL reagent for binding proteins in cell lysate than the corresponding TPD probe. In a similar study, the same group [81] synthesized a two-step clickable PAL probe (Fig. 28 Right) from anticancer saponin OSW-1 for the PAL study of its direct binding proteins in live cells. The OSW-1-based PAL probe retained potent anticancer activity, equivalent to that of the parent natural product, which allowed a cell-permeable analogue of OSW-1. The PAL studies demonstrated that the probe enabled crosslinking of a model sterol-binding protein in an affinity-dependent fashion, which can be easily detected by conjugation with a fluorophore or biotin via click chemistry. The photochemical and biological properties of the probe provide a platform for efficient capture and detection of proteins in their native environment.

**Fig. 29:**
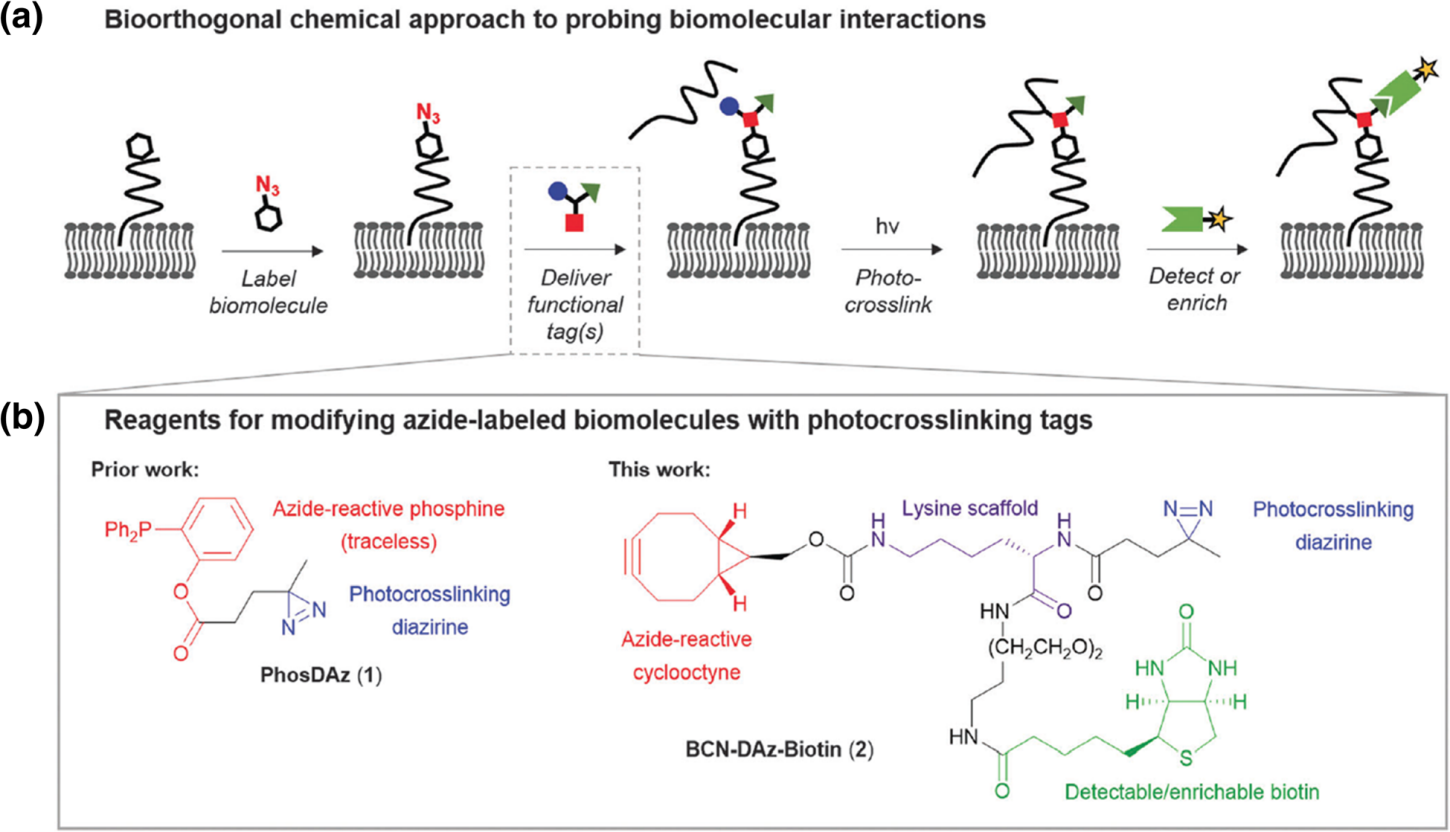
**a** Schematic representation of bioorthogonal chemistry approach for to biomolecular interactions. **b** Reagents for delivering photocrosslinking functionality to azide-labeled biomolecules, including the previously reported PhosDAz and the reagent reported herein, BCN-DAz-Biotin. Figure reproduced from ref. [82] with permission from RSC publication

A bioorthogonal chemical approach was also used to study PPIs. The Benjamin M. Swarts group [82] developed a novel bicyclo[6.1.0]nonyne (BCN)-based cyclooctyne with DA as a photocrosslinking group and a biotin affinity handle for pull-down, named BCN-DAz-Biotin (Fig. 29). The BCN-DAz-Biotin probe contains photocrosslinker (DA), SPAAC-based azide-labeling motif (cyclooctyne) for biomolecules, and biotin for enrichment/detection of interacting species in native contexts. They have demonstrated the utility of the BCN-DAz-Biotin probe with BSA. Furthermore, the use of this probe was demonstrated using cell surface azides in the bacterium M. smegmatis, proving its applicability in living systems. The authors envisioned that BCN-DAz-Biotin could assist the study of biomolecular interactions, specifically where strategies already exist for incorporating azides into the biomolecule of interest.

## Comparative probes

**Fig. 30:**
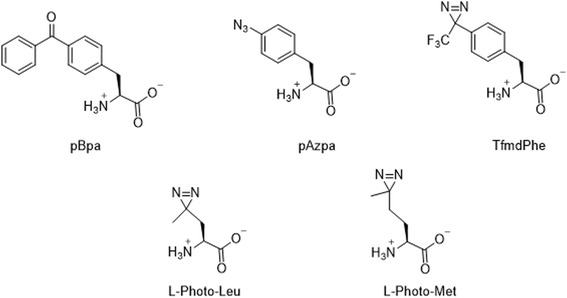
Structures of photocrosslinking amino acids that have been incorporated into cellular proteins

The breakthrough in the use of photocrosslinkers came when the Schultz and Yokoyama groups developed the photo-affinity-based genetically encoded amino acids (Fig. 30). These amino acids are based on three different photocrosslinkers (i.e., BP, AA, and DA) [23, 41–43, 83–95]. This unnatural amino acid mutagenesis technology enabled researchers to incorporate photocrosslinker site-specifically. In the next part of this review, we cover the comparative study of PLs. The Schultz group incorporated BP, AA, and trifluoromethylphenyl DA amino acids into proteins synthesized in E. coli. Peter E. Nielsen et al. used a similar method for site-specific incorporation of two novel bicyclic amino acids, benzofuranylalanine and benzotriazolylalanine, into E. coli proteins.

**Fig. 31:**
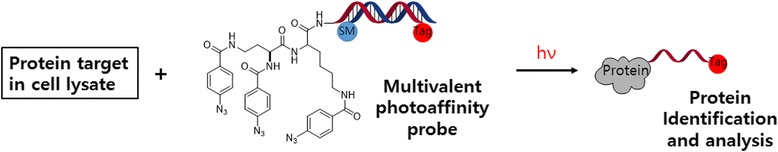
Schematic representation of multivalency approach

The multivalency effect was studied by Xiaoyu Li et al. [96], who screened a variety of crosslinkers. The crosslinking efficiency was improved by nearly 7-fold by using the multivalency effect (Fig. 31), without compromising probe specificity. The result of their initial screening reveals that simple, unsubstituted phenyl azide was the optimal photocrosslinker, mostly due to its tendency to generate longer-lived reactive intermediates. Further, they introduced multiple phenyl azide crosslinkers into the probe, which significantly improved the labeling yields. The major advantage of this DPAL (DNA-programmed affinity labeling) is that capture probe modification does not affect SM − protein binding; therefore, they could introduce four or more crosslinkers to further improve the labeling efficiency. The most important aspect of this method is that despite incorporating multiple photocrosslinkers, the probe specificity is not compromised. Overall, this type of multivalent probe may have wide application in studying small molecule − protein interactions with higher labeling yields and for sensitive protein detection when coupled with PCR amplification.

**Fig. 32:**
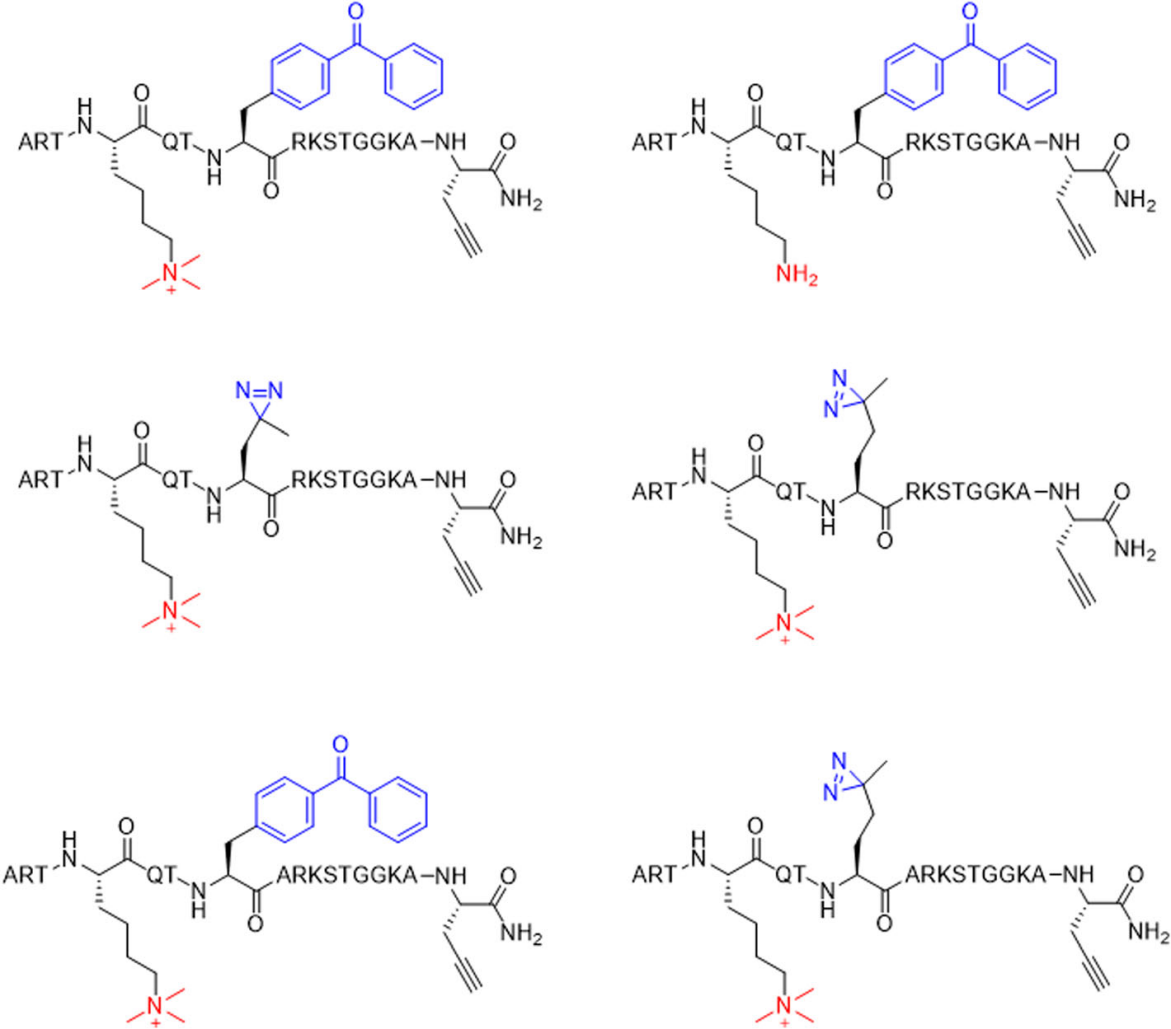
Chemical structures of photo-affinity probes 1–5 and C1

Some of the previous probes for histone PTMs [56] were compared with the new probes by Xiang David Li et al. [97] For this comparative study, the authors developed DA-based photo-affinity probes to capture the ‘readers’ of histone lysine methylation as well as the ‘erasers’ of histone lysine acetylation and malonylation (Fig. 32). They selected DA because it has several advantages for PAL, including small size, short lifetime upon UV irradiation and high reactivity. These new probes with DA demonstrated higher photocrosslinking efficiencies and specificities in the tested systems. An important point in the design of photo-affinity probes is to determine the position of the photoreactive group. DA, due to its small size, has more flexibility can be easily incorporated into any site, particularly closer to PTM sites. In contrast, due to bulky nature of BP, incorporation is not as easy. The incorporation of DA close to PTM site improved the efficiency and specific labeling of proteins that recognize PTMs. These crucial characteristics make DA a more suitable PAL agent than BP. Furthermore, the authors demonstrated that DA-based probes could also be used to capture lysine deacetylases and demalonylase. Thus, this methodology broadened the scope of our photocrosslinking strategy for the identification of histone PTM ‘readers’ to identifying dynamic and transient interactions between PTMs and their ‘erasers’.

**Fig. 33:**
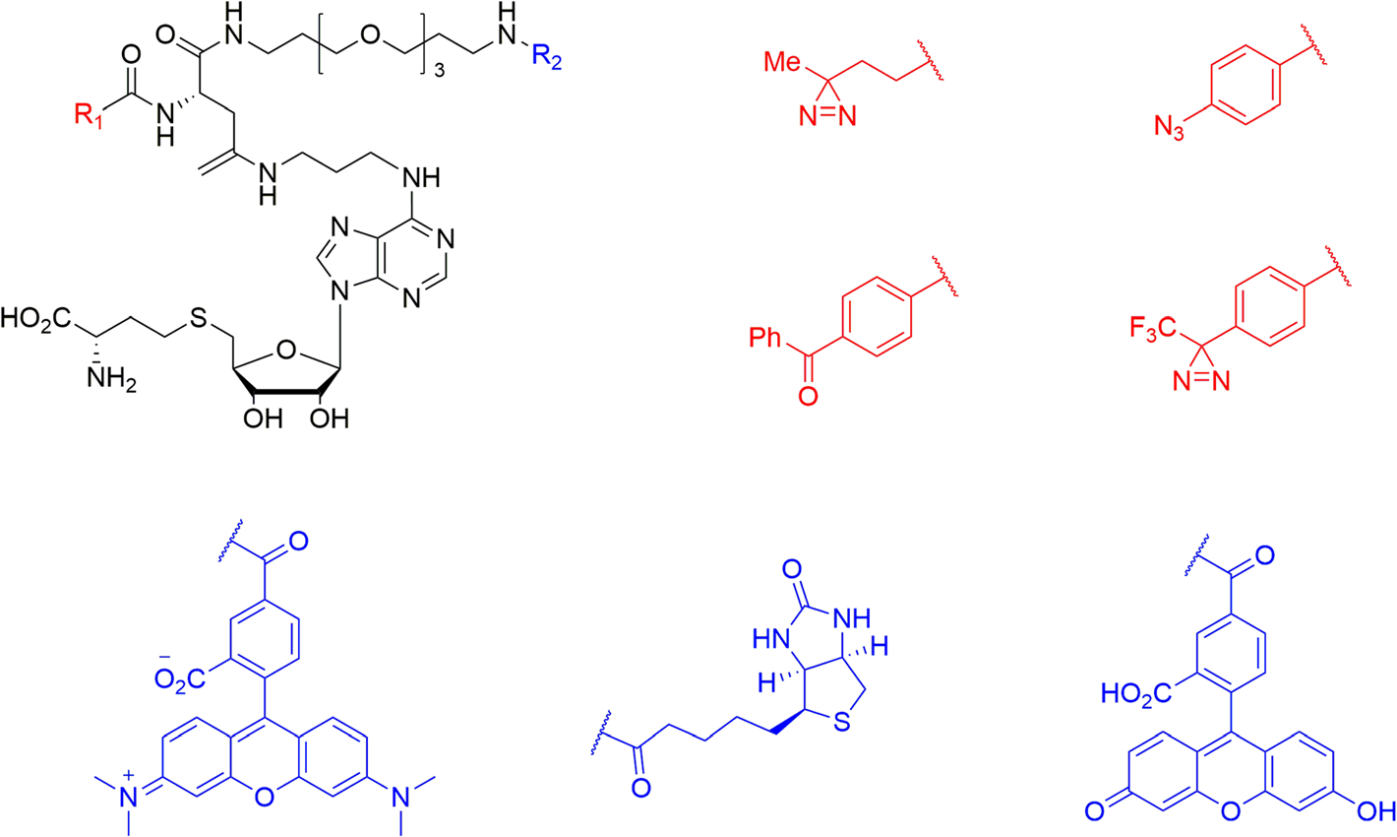
(SAH)-based photoreactive probes for chemical proteomic profiling of methyltransferases

Benjamin Cravatt et al. [98] described a set of S-adenosyl homocysteine (SAH) photoprobes (Fig. 33), that can be used in chemical proteomic experiments to profile and enrich a large number of MTs (methyltransferases) (>50) from human cancer cell lysates with high specificity over other classes of proteins. They further proved that SAH probes could enrich MT-associated proteins and be used to screen for and to assess the selectivity of MT inhibitors, which led to the discovery of a covalent inhibitor of nicotinamide N-methyltransferase (NNMT), an enzyme implicated in cancer and metabolic disorders. The chemical proteomics probes and methods for their utilization reported therein could be valuable for the functional characterization of MTs, MT complexes, and MT inhibitors in mammalian biology and disease. Their results emphasized that altering the identity of the photoreactive group itself may not improve the coverage of MTs, as replacing the DA with BP or AA groups produced probes that mainly targeted subsets of the MTs enriched by the DA probes.

**Fig. 34:**
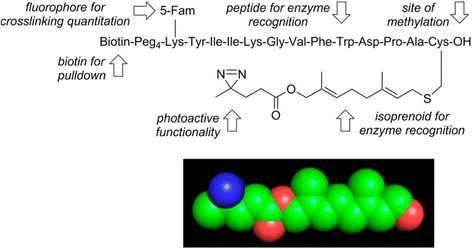
PAL based on Photoactivatable Isoprenoid. Figure reproduced from ref. [99] with permission from ACS publication

Incorporation of PLs into multifunctional peptides was studied by Mark D. Distefano et al. [99] They described the development and application of a new class of isoprenoid analogues with DA as the PAL moiety (Fig. 34). The designed photoactive farnesyl probes were synthesized in six steps and were finally incorporated into a multifunctional peptide produced via solid-phase synthesis. This multifunctional DA-containing peptide was a substrate for Ste14p. Next, they compared the probe with its BP counterpart. The result of this study showed that the DA-containing peptide was an efficient substrate for the enzyme. Additionally, the PAL efficiency of the DA probe was better than that of the BP probe. Finally, the crosslinked products were detected with the help of the incorporated fluorophore. The greater PAL yield of His-Ste14p coupled with the ease of analysis of this new class of photoprobe could be useful for the identification of active-site residues in His-Ste14p.

**Fig. 35:**
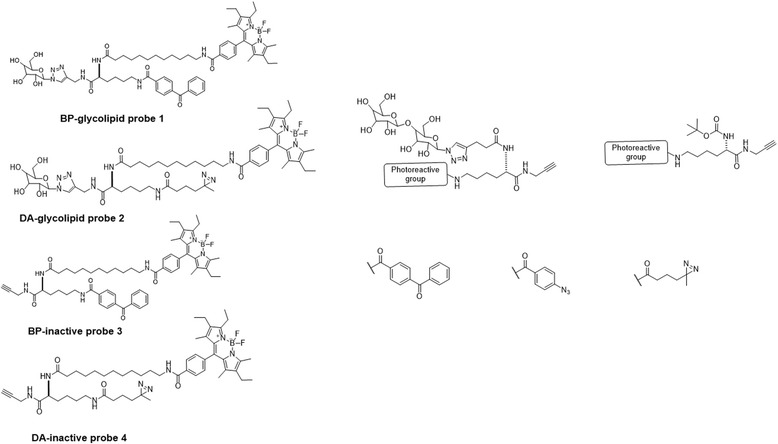
(*left*) Structures of glycolipid photo-affinity probes (1,2) with corresponding control probes (inactive probes) (3,4). (*Right*) Structures of lactose-based photo-affinity probes

Glycolipid probes were extensively studied by the Kaori Sakurai group [100] via the design and synthesis of trifunctional photo-affinity glycolipid probes (Fig. 35 left), with a sugar head group with a triazole linkage to the lipid tail unit containing a photoreactive group and a fluorescent tag. The glycolipid PAL probes with BP or DA groups were evaluated for their photocrosslinking reactivity toward a carbohydrate head group specific-protein. The PAL data showed the DA-based glycolipid photo-affinity probe was more effective than the BP-based probe in a comparative analysis involving a competitive ligand to distinguish a specific binding protein. Both probes displayed significant PAL reactivity toward nonspecific proteins due to the hydrophobicity of the lipid tail moiety. The researchers also evaluated two approaches to distinguish a specific binding protein by comparing with an inactive probe or a competitive ligand in parallel reactions. The comparative analysis involving a competitive ligand was more reliable, and the DA probe and enabled more straightforward detection of a specific carbohydrate-binding protein (i.e., b-glucosidase) than the BP probe. Overall, these experiments demonstrated that DA-based glycolipid photo-affinity probes are more suitable than BP-based probes to explore specific glycolipid binding proteins. The researchers [101] also compared the reactivity of carbohydrate PAL probes with different photoreactive groups (Fig. 35 Right). They designed and synthesized a set of carbohydrate-based PAL probes to compare the effects of different photoreactive groups (BP, DA, and AA) on the efficiency and selectivity of the PAL of a low-affinity binding protein. The PAL data proved that the DA probe gave low crosslinking yields but showed highly ligand-dependent reactivity via PAL. Later, the authors observed different results with different photoreactive groups for PAL experiments in the cell lysate. Finally, they showed that the DA-based probe was highly selective for crosslinking a low-affinity binding protein, which could not be achieved when AA or BP groups were used.

**Fig. 36:**
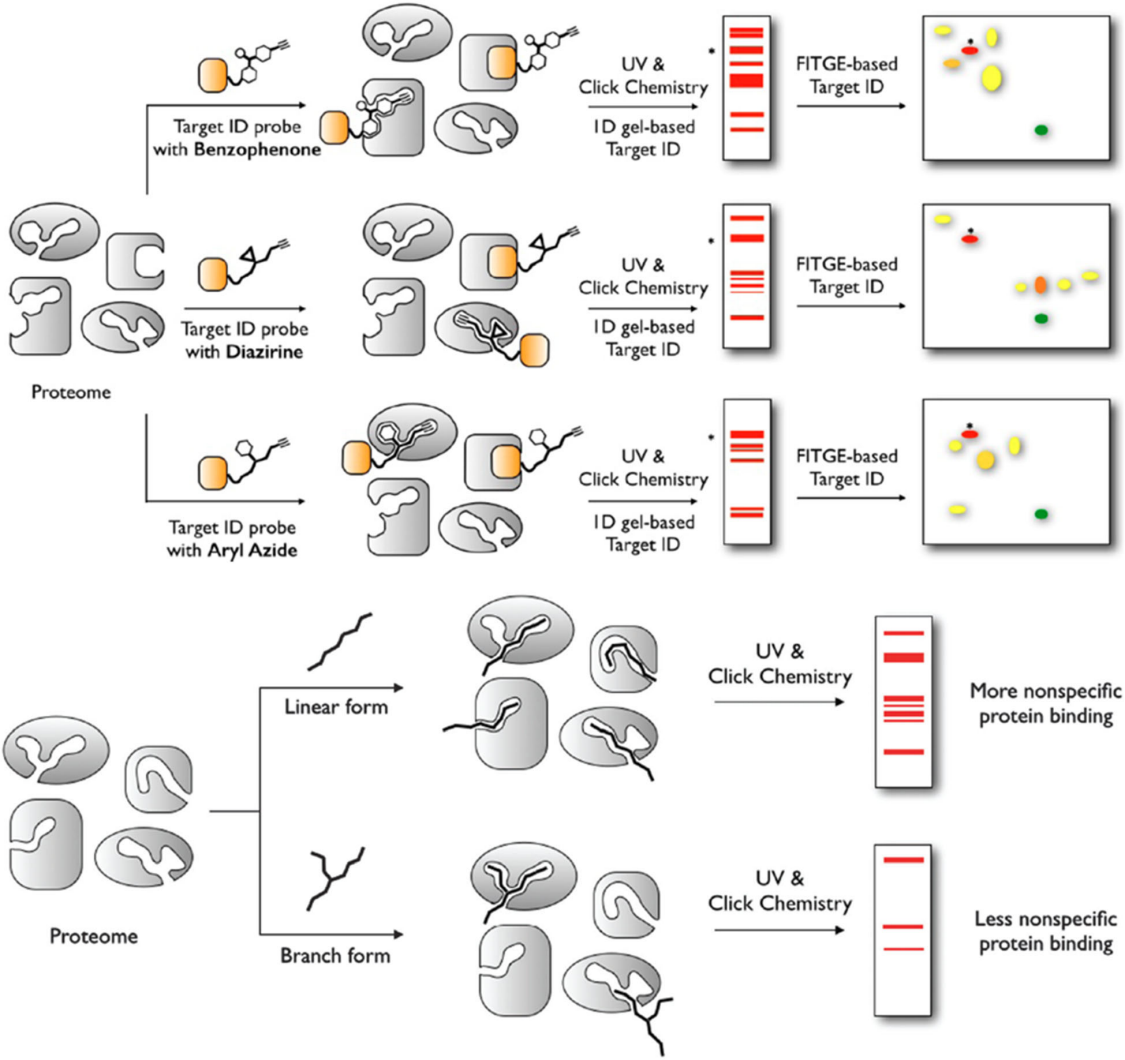
(*top*) Schematic of photo-affinity-based target identification (ID) with different photoactivatable linkers. Each target ID probe containing a photoactivatable moiety (BP, DA and AA) which can bind to a specific set of proteins in a structure-dependent manner. Figure reproduced from ref. [102] with permission from ACS publication. (*Bottom*) Schematic illustration of the molecular shape-dependence of protein labeling. The flexibility of linear molecules increases the binding to various proteins. Branched molecules bind to fewer proteins than linear molecules due to their restricted conformational flexibility. Figure reproduced from ref. [103] with permission from RSC publication

The structure-dependent approach of PLs to study PPIs was developed by the Seung Bum Park group (Fig. 36 top). They reported [102] the PAL of photo-affinity linkers containing three photoactivatable moieties, DA, BP and AA. They found that each photo-affinity group binds to a different set of proteins in a structure-dependent manner, in contrast to previous beliefs. They obtained the complete list of proteins labeled by each photo-affinity linker, which was used to eliminate the nonspecific binding proteins from the target candidates, ultimately increasing the success rate of target identification. They also proposed that the target protein-labeling pattern is not associated with the labeling efficiency of PLs (AA > DA > BP). Based on this PL labeling efficiency, AA and DA might be good choices for target protein labeling, but the target ID results showed that DA was not a good choice as a PL of the target ID probe. This systematic protein-labeling pattern analysis of PLs can be used to discriminate a target protein from specific nontarget proteins encountered in the actual target ID process. The researchers also demonstrated [103] the importance of PL in the design of target ID probes using three case studies. These studies strongly suggested that target proteins might be missed if an appropriate PL is not chosen for the target ID probe. Although they were not able to suggest the best PL, they stated that in order to encounter a POI, at least two types of PLs are required for a successful target ID process. The same group reported a molecular shape-dependent approach for nonspecific labeling of photo-affinity linkers (PLs) in the cellular proteome (Fig. 36 bottom). In this report, they compared five types of PAL reagents with various molecular shapes and different photoactivatable moieties. The PAL data showed a significant reduction in nonspecific protein labeling by branched PLs compared with linear PLs in living cells. This may be due to the high conformational flexibility of linear PLs. These data supported the use of branched PLs in specific labeling procedures to avoid nonspecific binding. They also identified a smaller branched DA-based PL as the best photo-affinity probe for PAL. As a proof-of-principle, they synthesized a tubulin-selective photo-affinity probe and demonstrated that the well-designed probe plays a vital role in identifying target proteins in living cells.

**Fig. 37:**
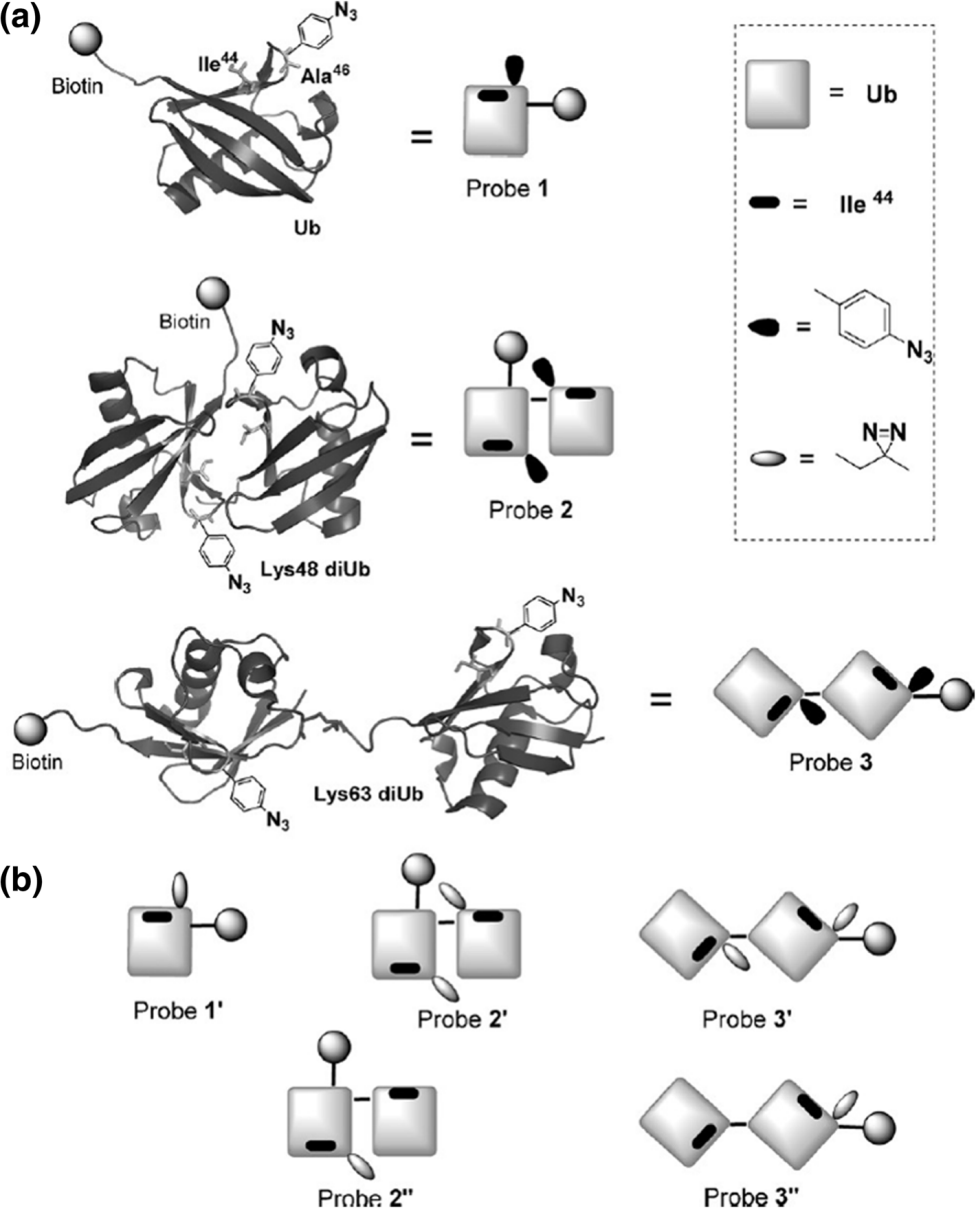
Photo-affinity probes with crosslinking groups attached to Ala46 **a**) Phenyl-azide-based ubiquitin probes **b**) The diazirine-based ubiquitin probes

Chang-Lin Tian et al. [104] described the chemical synthesis of diubiquitin-based photo-affinity probes for the identification ubiquitin-binding proteins (Fig. 37). DA-based photo-affinity probes were used to capture Ub-binding proteins in cell lysates. This PAL study also showed that DAs are preferable to AAs as the photocrosslinking group due to the comparatively lower nonspecific capture. Additionally, they demonstrated at least two Ub units were required to effectively capture Ub-binding proteins. The selectivity varied for different types of linkages containing diubiquitin moieties, indicating the importance of linkage-dependent probes to selectively profile Ub-binding proteins under different cellular conditions.

**Fig. 38:**
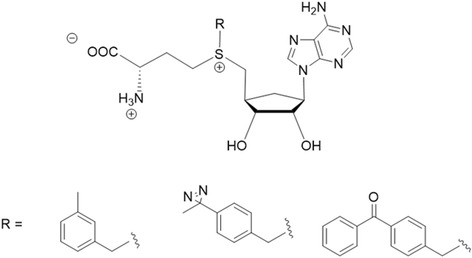
Chemical structure of novel AdoMet analogues with photo-cross-linking side chains

Andrea Rentmeister et al. [105] recently reported three novel S-adenosyl-l-methionine (AdoMet) analogues bearing the most common photocrosslinking moieties (AA, DA, and BP) (Fig. 38). These new AdoMet probes can be used as tools for the enzymatic transfer of photocrosslinkers to identify RNA-protein interactions. Enzymes are used to transfer the photocrosslinking moieties to the N7 position of the mRNA cap with high efficiency. The PAL study showed that the DA-and AA-modified cap analogues retained the ability to bind to the cap binding protein eIF4E, whereas the BP-modified analogue did not bind. Since the wavelength required for photocrosslinking is longer for DA than AA, it less damaging to AdoMet. Thus, the probe with DA is the best choice for enzymatic transfer and photocrosslinking to a directly interacting protein.

**Fig. 39:**
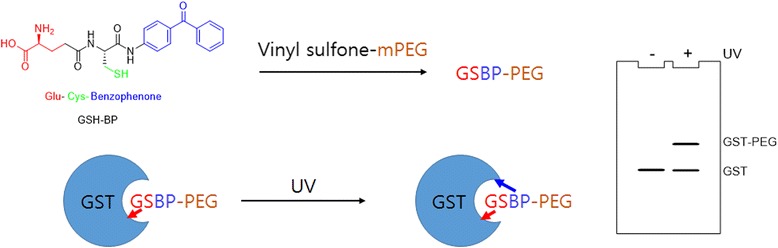
Schematic overview of the photo-affinity PEGylation using GSH-BP

Protein − polymer conjugation-based probes have been used to study the binding affinity of GSH to GST. Heather D. Maynard et al. [106] designed and chemically synthesized a GSH analogue (GSH-BP) with three functionalities: (1) the binding affinity of GSH to GST, (2) a free thiol for polymer functionalization, and (3) a photoreactive BP (BP) component (Fig. 39). Different PEGs with different MWs have been used (2 kDa, 5 kDa, and 20 kDa) to synthesize GSH-BP-modified PEGs (GSBP-PEGs), and the PAL studies showed conjugation efficiencies between 52 and 76% to GST. To better understand the PAL regent with DA, PEG was also prepared, which compared with the BP-based probe, produced lower conjugation yields. To validate the utility of each component of the design, PEGs with different end-groups, including glutathione (GS-PEG) and BP (BP-PEG), were synthesized. The PAL study showed that GSH and BP were both crucial for successful conjugation to GST. Finally, the specific binding was confirmed with the conjugation of 5 kDa GSBP-PEG to different proteins, including bovine serum albumin (BSA), lysozyme (Lyz), ubiquitin (Ubq), and GST-fused ubiquitin (GST-Ubq). Overall, a new phototriggered protein − polymer conjugation method that is generally applicable for the identification of GST-fusion proteins was developed.

**Fig. 40:**
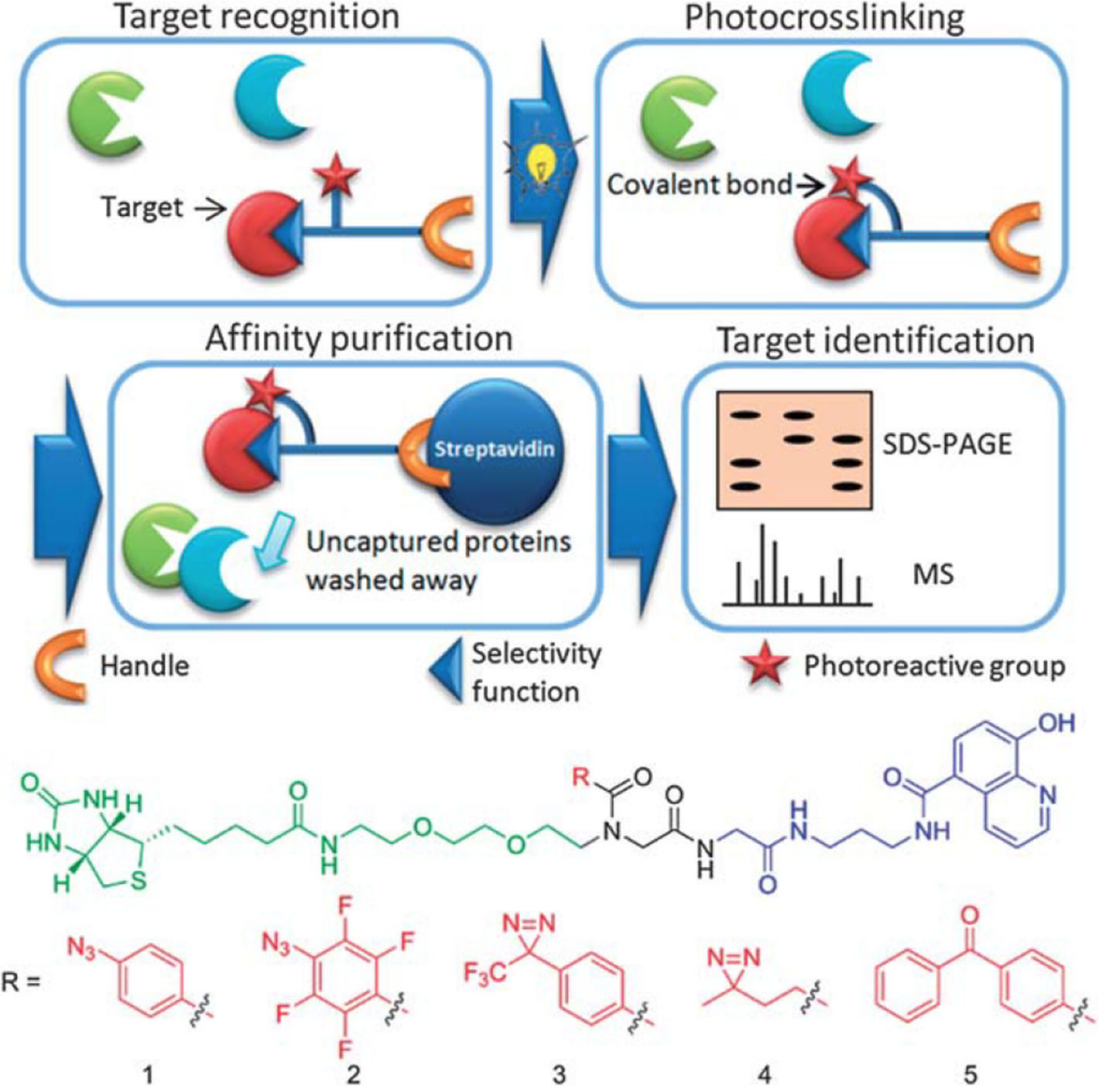
schematic representation of the application of photo-affinity probes with potential photo-affinity probes for 2-oxoglutarate oxygenases incorporating 5 different photoreactive groups. Figure reproduced from ref. [107] with permission from RSC publication

Difficulty in the synthesis of photo-affinity probes is a major issue in the development of probes. To address this issue, Christopher J. Schofield et al. [107] demonstrated the Ugi four-component reaction for efficient synthesis and comparison of PAL probes (Fig. 40). The photo-affinity probes consisted of a photo-affinity group, detection handles, and inhibitor attachment points. The synthesized set of photo-affinity probes showed affinity for 2-OG oxygenases. The authors reported the photocrosslinking rates, yields and sites using PHD2 as a model system. The PAL study reveals the substantial differences between probes. AA and alkyl DA-based probes showed better crosslinking efficiency, while aryl trifuoromethyl DA and BP probes gave low crosslinking yields. The lower efficiency with trifluoromethyl phenyl DAs and BP indicated that the optimum photoreactive group differs depending on the ‘intrinsic’ photochemical properties of the probe and the nature of its interaction with the target protein. The other factors in achieving a better crosslinking yield are the proximity and orientation of the photoreactive group and the rate of reaction with the enzyme versus the quenching reaction.

**Fig. 41:**
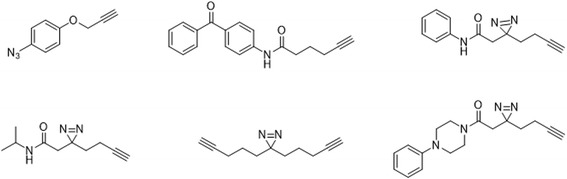
Photocrosslinker probes used in this study. The photoreactive group are AA, BP and DA

A comprehensive inventory development-based approach was used by Sieber group [108] to investigate background protein labeling by gel-free quantitative proteomics. Specific off-targets were identified for each photoreactive group and were accumulated in a comprehensive inventory. The researchers compared known photocrosslinkers (AA, DA, and BP) (Fig. 41). AA and DA were found to be superior to BP, in agreement with previous experiments comparing crosslinking efficiencies. The comparison of four DA probes revealed a common set of false positives. The most prominent protein hits were proteins with high abundance with a preference for small-molecule binding. Additionally, the PAL was dependent on the linker length. Probes with small linkers and aliphatic DAs resulted in the least binding, whereas aromatic substituents significantly increased the number of hits. In a proof-of-principle study, a DA moiety was attached to H8, a protein kinase A inhibitor. The PAL study of this probe provided insight into its in situ proteome targets. Additionally, the authors demonstrated successful target identification and the necessity for a photocrosslinker-specific exclusion list.

## Tetrazole-based probes as PAL reagents

**Fig. 42:**
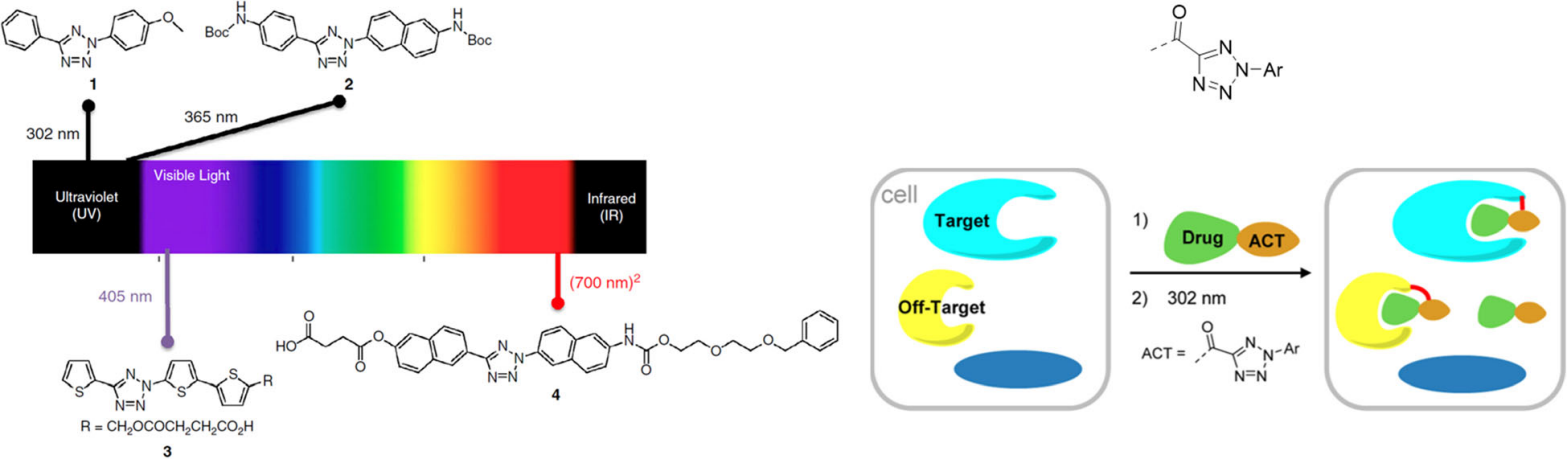
(*Left*) Design of tetrazoles with variable photoactivation wavelengths. Figure reproduced from ref. [109] with permission from ScienceDirect publication. (*Right*) tetrazole as a New Photo-affinity Label for Drug Target Identification. Figure reproduced from ref. [110] with permission from ACS publication

Qing Lin designed tetrazole reagents [109] that can be activated using a biocompatible light source (Fig. 42 left). The main features of the design are the robustness, genetically encodable alkene reporters, and the improved understanding of the reaction mechanism. Additionally, they reported that tetrazole-alkene photoclick chemistry could play a major role as a reactivity-based tool in biological systems. They outlined the recent developments in the optimization of ‘photoclick chemistry’. The points they considered are the need for a substrate that offers two-photon photoactivatability, superior reaction kinetics, and/or genetic encodability to study the reaction mechanism. Additionally, they demonstrated the application of photoclick chemistry in in vitro and in vivo protein labeling, as well as in preparing ‘smart’ hydrogels for 3D cell culture. The potential applications of photoclick chemistry may include multiplexed analysis of glycan dynamics with tetrazole ligation. Furthermore, the inherent ‘turn-on’ fluorescence property makes photoclick chemistry useful for ‘no-wash’ fluorescent labeling.

In continuation of their primary investigation of tetrazole as a photocrosslinker, the researchers developed a novel PAL reagent, called 2‑Aryl-5-carboxytetrazole (ACT) [110], for drug target identification (Fig. 42 Right). They reported a unique mechanism of this ACT probe, in which the photogenerated carboxynitrile imine reacts with a proximal nucleophile near the target active site. In contrast, traditional photocrosslinkers work through nonspecific C − H/X − H bond insertion reactions with the POI via a highly reactive intermediate. As a proof-of-concept study, they selected dasatinib and JQ-1 as the desired targets. Compared with the most commonly used PAL reagents, such as DA and BP, ACT showed high photocrosslinking yields toward their protein targets in vitro based on mass spectrometry analysis. In the in situ target identification studies, ACT successfully captured the desired targets with an efficiency comparable to that of DA.

**Fig. 43:**
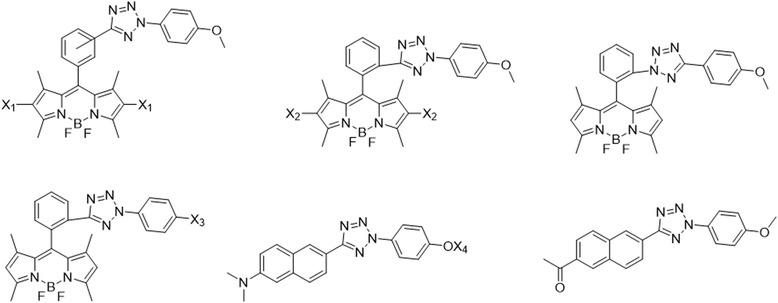
Structures of tetrazole-containing one- and two-photon probes based on Bodipy and Acedan dyes, respectively

Shao Q. Yao et al. recently studied tetrazole photoclick chemistry for PAL [111]. As in previous studies, they addressed photolysis of a tetrazole that generates a highly reactive nitrile imine, which undergoes rapid nucleophilic reaction with neighboring nucleophiles in a biological system. They also reported the expected cycloaddition with alkenes. To validate the application of the tetrazole photoclick reaction, different probes were synthesized based on BODIPY and Acedan dyes (Fig. 43). The researchers used fluorescent dyes conjugated with tetrazole to study novel photocrosslinkers with one- and two-photon fluorescence turn-on properties that were developed into protein-detecting biosensors. The no-wash imaging of the endogenous kinase activity was the reason behind the use of the fluorescent dyes. The authors are hopeful that this novel approach will find a wide range of applications in chemical biology to study PPIs.

## Quantitative proteomics to study protein-protein interactions

**Fig. 44:**
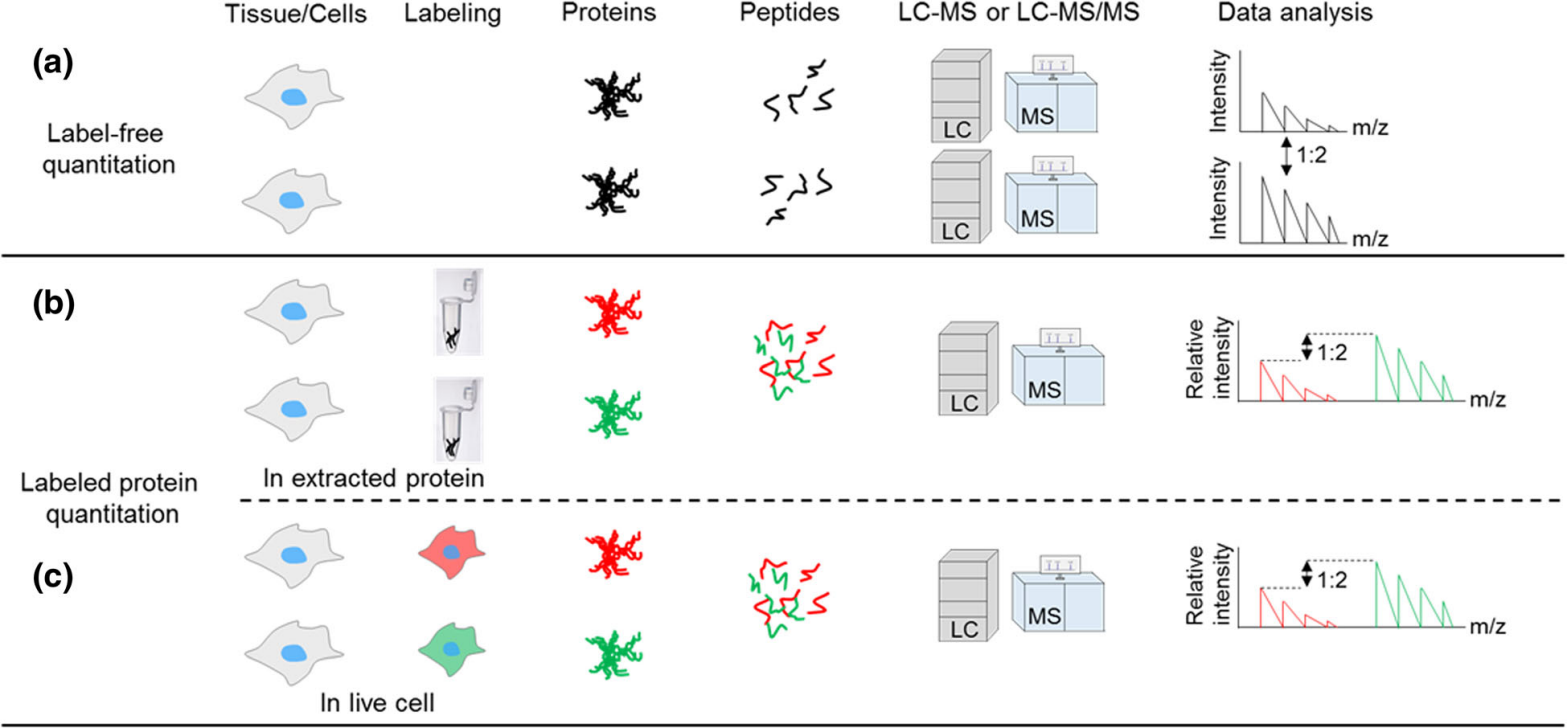
Schematic outlines showing typical workflows for quantitative proteomics from cells or tissues (from protein extraction, trypsin digestion and/or isotope labeling to MS analysis). Label-free quantitation individually analyzes samples and compares the data using multiple approaches like spectral counting and peak intensities. As unlabeled samples are individually analyzed in label-free workflows, the steps must be tightly controlled to avoid biasness. Conversely, labeled protein quantification is characterized by the isotopic labeling of proteins either after protein extraction or in live cell condition. Then, the labeled samples are combined and processed for quantitative analysis. The red and the green colors represent heavy and light isotopes, respectively, for differential labeling and comparison

Quantitative proteomics is the differential study of the abundance of proteins from distinct biological samples. Absolute proteomic quantification utilizes standard peptides of series concentrations. The spectral counts of the target peptide in the sample were compared to the known concentration standard peptide to calculate absolute concentration. On the other hands, relative quantification can be performed by comparisons of spectral counts among the samples. In label-free quantification, the proteins from two different samples are separately analyzed by MS and compared with respect to the spectral counting and peak intensities to determine the protein quantities in the samples (Fig. 44a). However, the drawback of this approach is the lack of internal standards.

Chemical tag labeling for relative quantitation, such as isotope-coded affinity tags (ICAT), is one strategy to improve proteome-wide quantitation by labeling the samples with MS isotopes to distinguish proteins from different samples (Fig. 44b) [112, 113]. Since the first report of ICAT, similar tagging approaches has been further developed, include tandem mass tags (TMTs) [114], isobaric tags for relative and absolute quantification (iTRAQ) [115], and dimethyl labeling [116, 117]. ICAT mainly uses two reagents with heavy and light isotopes in two different samples to label whole proteomes. The most commonly used isotopes are carbon (^13^C_6_/^12^C_6_), hydrogen (^2^H_1_/^1^H_1_), oxygen (^18^O_8_/^16^O_8_), nitrogen (^15^N_7_/^14^N_7_), iodine (^127^I_53_/^126^I_53_). The extracted proteomes from the samples are digested, and isotopes are incorporated into the digested peptides to produce a known mass shift in the MS. Differentially labeled samples are combined, and the differences in the mass peaks of the isotopes are analyzed to reflect the differences in the abundance of the corresponding proteins. The labeling approach is the most diverse due to the covalent chemical labeling [118].

An alternative labeling strategy for quantitative proteomics is the use of stable isotope labeling by/with amino acids in cell culture (SILAC) [119–122], which facilitates the labeling of the protein when it is synthesized in the cell (Fig. 44c) [123]. In this method, mammalian cells are maintained in a growth medium, which contains non-radioactive and isotopically labeled form of amino acids [124]. Double-labeling is also conceivable to ensure the almost ideal labeling of all peptides, for example Arg/Lys (^12^C_6_/^13^C_6_-arginine/^12^C_6_/^13^C_6_-lysine) [121].

By applying these quantitative proteomics techniques, photo-affinity tags can lead to the discoveries of unknown PPI or small molecule’s off-targets. To maximize the merit of photo-affinity labeling in intact endogenous condition, it should be emphasized to design quantitative proteomic experiment carefully to obtain valid interactions.

## Conclusions

The main objective of this review is to present a detailed description of protein-protein interactions using photo-affinity labeling. In this review, we try to cover most of the recently developed PAL agents. The PAL agents are mainly based on diazirine (alkyl and aryl), aryl azide and benzophenone as photocrosslinkers. For the detailed study and ease of reading the article, we have divided the article into different classes based on the PLs. The main outcome that we want to emphasize is that there is no universal PL scaffold. Many researchers have used different PLs for detailed investigations, and each researcher has identified a different PL as the best, possibly because each PL has its own distinct properties and the superiority of a PL depends upon the POI. Several research group have found that the size and flexibility of the PL are important criteria; the smaller the size is, the better the PL. In this regard, most of the studies report that DA is better than the others as it is very easy to incorporate DA-based probes near the active site. Another research groups have found that the chain length of the PL from the fluorophore is an important factor. Additionally, some studies have found that the shape of the linker matters, as branched chains are better than linear chains. It is also found that the abundance of the POI is another factor to consider when they choose the PL. In some cases, the wavelength required for photocrosslinking also makes a difference, e.g., in the case of DA, the wavelength is longer than that of AA; thus DA performed better than AA as it was less damaging to the POI. Probes with more than one PL in their structure (multivalent probes) are more useful photoprobes than those with only one PL with respect to photocrosslinking efficiency. Based on these case studies, it is recommended to examine series of photo-crosslinkers for each study to identify relevant PPI, and there is no universal tool to investigate endogenous PPI at this moment. It is also noteworthy that proper quantitative proteomic technique should be incorporated with PAL for successful intact PPI research. Careful selection of quantitative proteomic profiling together with PAL screening may lead to the discoveries of new biomarkers and novel therapeutic pathways.

## Abbreviations

2-DE: 2-dimensional gel electrophoresis
AA: Aryl azide
ABBP: Activity-based protein profiling
BP: Benzophenone
Bpa: p-benzoylphenylalanine
DA: Diazirine
GFP: Green fluorescence protein
ICAT: Isotope-coded affinity tags
iTRAQ: Isobaric tag for relative and absolute quantification
LC-MS: Liquid chromatography/mass spectrometry
PAL: Photo-affinity labeling
Pls: Photocrosslinkers
POI: Protein of interest
PPI: Protein-protein interaction
PPI: Protein-protein interactions
PTM: Post-translational modification
SILAC: Stable isotope labeling by/with amino acids in cell culture
TMT: Tandem mass tag
TPD: Trifluoromethylphenyl DA

## References

[1] Jin L, Wang W, Fang G (2014). Targeting protein-protein interaction by small molecules. Annu Rev Pharmacol Toxicol.

[2] Lowder MA, Appelbaum JS, Hobert EM, Schepartz A (2011). Visualizing protein partnerships in living cells and organisms. Curr Opin Chem Biol.

[3] Tsien RY (1998). The green fluorescent protein. Annu Rev Biochem.

[4] Zhang J, Campbell RE, Ting AY, Tsien RY (2002). Creating new fluorescent probes for cell biology. Nat Rev Mol Cell Biol.

[5] Andresen M, Schmitz-Salue R, Jakobs S (2004). Short tetracysteine tags to β-tubulin demonstrate the significance of small labels for live cell imaging. Mol Biol Cell.

[6] Griffin BA, Adams SR, Tsien RY (1998). Specific covalent labeling of recombinant protein molecules inside live cells. Science.

[7] Martin BR, Giepmans BN, Adams SR, Tsien RY (2005). Mammalian cell-based optimization of the biarsenical-binding tetracysteine motif for improved fluorescence and affinity. Nat Biotechnol.

[8] Los GV, Encell LP, McDougall MG, Hartzell DD, Karassina N, Zimprich C, Wood MG, Learish R, Ohana RF, Urh M (2008). HaloTag: a novel protein labeling technology for cell imaging and protein analysis. ACS Chem Biol.

[9] Keppler A, Gendreizig S, Gronemeyer T, Pick H, Vogel H, Johnsson K (2003). A general method for the covalent labeling of fusion proteins with small molecules in vivo. Nat Biotechnol.

[10] Gautier A, Juillerat A, Heinis C, Corrêa IR, Kindermann M, Beaufils F, Johnsson K (2008). An engineered protein tag for multiprotein labeling in living cells. Chem Biol.

[11] Chen I, Howarth M, Lin W, Ting AY (2005). Site-specific labeling of cell surface proteins with biophysical probes using biotin ligase. Nat Methods.

[12] Howarth M, Takao K, Hayashi Y, Ting AY (2005). Targeting quantum dots to surface proteins in living cells with biotin ligase. Proc Natl Acad Sci U S A.

[13] Martell JD, Deerinck TJ, Sancak Y, Poulos TL, Mootha VK, Sosinsky GE, Ellisman MH, Ting AY (2012). Engineered ascorbate peroxidase as a genetically encoded reporter for electron microscopy. Nat Biotechnol.

[14] Calloway NT, Choob M, Sanz A, Sheetz MP, Miller LW, Cornish VW (2007). Optimized fluorescent trimethoprim derivatives for in vivo protein labeling. ChemBioChem.

[15] Cai Y, Rossier O, Gauthier NC, Biais N, Fardin M-A, Zhang X, Miller LW, Ladoux B, Cornish VW, Sheetz MP (2010). Cytoskeletal coherence requires myosin-IIA contractility. J Cell Sci.

[16] Goldsmith CR, Jaworski J, Sheng M, Lippard SJ (2006). Selective labeling of extracellular proteins containing polyhistidine sequences by a fluorescein-nitrilotriacetic acid conjugate. J Am Chem Soc.

[17] Lata S, Gavutis M, Tampé R, Piehler J (2006). Specific and stable fluorescence labeling of histidine-tagged proteins for dissecting multi-protein complex formation. J Am Chem Soc.

[18] Ojida A, Honda K, Shinmi D, Kiyonaka S, Mori Y, Hamachi I (2006). Oligo-Asp tag/Zn (II) complex probe as a new pair for labeling and fluorescence imaging of proteins. J Am Chem Soc.

[19] Dormtin G, Prestwich GD (1994). Perspectives in Biochemistry.

[20] Kotzyba‐Hibert F, Kapfer I, Goeldner M (1995). Recent trends in photo-affinity labeling. Angew Chem Int Ed Engl.

[21] Prestwich GD, Dormán G, Elliott JT, Marecak DM, Chaudhary A (1997). BP photoprobes for phosphoinositides, peptides and drugs. Photochem Photobiol.

[22] Hashimoto M, Hatanaka Y (2008). Recent Progress in DA‐Based Photo-affinity Labeling. Eur J Org Chem.

[23] Tanaka Y, Bond MR, Kohler JJ (2008). Photocrosslinkers illuminate interactions in living cells. Mol BioSyst.

[24] Moerke NJ (2009). Fluorescence polarization (FP) assays for monitoring peptide‐protein or nucleic acid‐protein binding. Current Protoc Chem Biol.

[25] Gubbens J, de Kroon AI (2010). Proteome-wide detection of phospholipid-protein interactions in mitochondria by photocrosslinking and click chemistry. Mol BioSyst.

[26] Das J (2011). Aliphatic DAs as photo-affinity probes for proteins: recent developments. Chem Rev.

[27] Lenz T, Fischer JJ, Dreger M (2011). Probing small molecule-protein interactions: A new perspective for functional proteomics. J Proteome.

[28] Dubinsky L, Krom BP, Meijler MM (2012). DA based photo-affinity labeling. Bioorg Med Chem.

[29] Park J, Koh M, Park SB (2013). From noncovalent to covalent bonds: a paradigm shift in target protein identification. Mol BioSyst.

[30] Pham ND, Parker RB, Kohler JJ (2013). Photocrosslinking approaches to interactome mapping. Curr Opin Chem Biol.

[31] Preston GW, Wilson AJ (2013). Photo-induced covalent cross-linking for the analysis of biomolecular interactions. Chem Soc Rev.

[32] Sumranjit J, Chung SJ (2013). Recent advances in target characterization and identification by photo-affinity probes. Molecules.

[33] Xia Y, Peng L (2013). Photoactivatable lipid probes for studying biomembranes by photo-affinity labeling. Chem Rev.

[34] Tomohiro T, Hatanaka Y (2014). DA-Based Multifunctional Photo-Probes for Affinity-Based Elucidation of Protein-Ligand Interaction. Heterocycles.

[35] Sakurai K (2015). Photo-affinity Probes for Identification of Carbohydrate‐Binding Proteins. Asian J Org Chem.

[36] Smith E, Collins I (2015). Photo-affinity labeling in target-and binding-site identification. Future Med Chem.

[37] Dormán G, Nakamura H, Pulsipher A, Prestwich GD (2016). The Life of Pi Star: Exploring the Exciting and Forbidden Worlds of the BP Photophore. Chem Rev.

[38] Yang Y, Song H, Chen PR (2016). Genetically encoded photocrosslinkers for identifying and mapping protein‐protein interactions in living cells. IUBMB Life.

[39] Singh A, Thornton ER, Westheimer F (1962). The photolysis of diazoacetylchymotrypsin. J Biol Chem.

[40] Galardy RE, Craig LC, Jamieson JD, Printz MP (1974). Photo-affinity labeling of peptide hormone binding sites. J Biol Chem.

[41] Chin JW, Martin AB, King DS, Wang L, Schultz PG (2002). Addition of a photocrosslinking amino acid to the genetic code of Escherichia coli. Proc Natl Acad Sci.

[42] Chin JW, Schultz PG (2002). In vivo photocrosslinking with unnatural amino acid mutagenesis. Chembiochem.

[43] Farrell IS, Toroney R, Hazen JL, Mehl RA, Chin JW (2005). Photo-cross-linking interacting proteins with a genetically encoded BP. Nat Methods.

[44] Tantama M, Lin W-C, Licht S (2008). An activity-based protein profiling probe for the nicotinic acetylcholine receptor. J Am Chem Soc.

[45] Wittelsberger A, Mierke DF, Rosenblatt M (2008). Mapping Ligand-receptor Interfaces: Approaching the Resolution Limit of BP‐based Photo-affinity Scanning. Chem Biol Drug Des.

[46] Dugan A, Majmudar CY, Pricer R, Niessen S, Lancia JK, Fung HY-H, Cravatt BF, Mapp AK (2016). Discovery of Enzymatic Targets of Transcriptional Activators via in Vivo Covalent Chemical Capture. J Am Chem Soc.

[47] Saghatelian A, Jessani N, Joseph A, Humphrey M, Cravatt BF (2004). Activity-based probes for the proteomic profiling of metalloproteases. Proc Natl Acad Sci U S A.

[48] Rowland MM, Bostic HE, Gong D, Speers AE, Lucas N, Cho W, Cravatt BF, Best MD (2011). Phosphatidylinositol (3, 4, 5)-Trisphosphate Activity Probes for the Labeling and Proteomic Characterization of Protein Binding Partners. Biochemistry.

[49] Hilbold B, Perrault M, Ehret C, Niu S-L, Frisch B, Pécheur E-I, Bourel-Bonnet L (2011). BP-containing fatty acids and their related photosensitive fluorescent new probes: Design, physico-chemical properties and preliminary functional investigations. Bioorg Med Chem.

[50] Kawamura A, Hindi S, Mihai DM, James L, Aminova O (2008). Binding is not enough: flexibility is needed for photocrosslinking of Lck kinase by BP photoligands. Bioorg Med Chem.

[51] Wu Y, Olsen LB, Lau YH, Jensen CH, Rossmann M, Baker YR, Sore HF, Collins S, Spring DR (2016). Development of a Multifunctional BP Linker for Peptide Stapling and Photo-affinity Labeling. ChemBioChem.

[52] Marcon L, Wang M, Coffinier Y, Le Normand F, Melnyk O, Boukherroub R, Szunerits S (2009). Photochemical immobilization of proteins and peptides on BP-terminated boron-doped diamond surfaces. Langmuir.

[53] Tal-Gan Y, Naveh S, Klein S, Moshel O, Levitzki A, Gilon C (2012). Studying protein-peptide interactions using BP units: A case study of protein kinase B/Akt and its inhibitor PTR6154. Anal Biochem.

[54] Guo L-W, Hajipour AR, Gavala ML, Arbabian M, Martemyanov KA, Arshavsky VY, Ruoho AE (2005). Sulfhydryl-reactive, cleavable, and radioiodinatable BP photoprobes for study of protein − protein interaction. Bioconjug Chem.

[55] Li X, Foley EA, Molloy KR, Li Y, Chait BT, Kapoor TM (2012). Quantitative chemical proteomics approach to identify posttranslational modification-mediated protein-protein interactions. J Am Chem Soc.

[56] Li X, Kapoor TM (2010). Approach to profile proteins that recognize post-translationally modified histone ‘tails’. J Am Chem Soc.

[57] Sakurai K, Tawa M, Okada A, Yamada R, Sato N, Inahara M, Inoue M (2012). Active/Inactive Dual‐Probe System for Selective Photo-affinity Labeling of Small Molecule‐Binding Proteins. Chem-Asian J.

[58] Sakurai K, Yamada R, Okada A, Tawa M, Ozawa S, Inoue M (2013). Selective Fluorescence Detection of Small‐Molecule‐Binding Proteins by Using a Dual Photo-affinity Labeling System. ChemBioChem.

[59] Sakurai K, Hatai Y, Okada A (2016). Gold nanoparticle-based multivalent carbohydrate probes: selective photo-affinity labeling of carbohydrate-binding proteins. Chem Sci.

[60] Murale DP, Hong SC, Yun J, Yoon CN, Lee J-S (2015). Rational design of a photo-crosslinking BODIPY for in situ protein labeling. Chem Commun.

[61] Hw A, Shen W, Sagi A, Chen PR, Schultz PG (2011). Probing Protein-Protein Interactions with a Genetically Encoded Photo‐crosslinking Amino Acid. Chembiochem.

[62] Chou C, Uprety R, Davis L, Chin JW, Deiters A (2011). Genetically encoding an aliphatic DA for protein photocrosslinking. Chem Sci.

[63] Lin S, He D, Long T, Zhang S, Meng R, Chen PR (2014). Genetically encoded cleavable protein photo-cross-linker. J Am Chem Soc.

[64] Yang Y, Song H, He D, Zhang S, Dai S, Lin S, Meng R, Wang C, Chen PR (2016). Genetically encoded protein photocrosslinker with a transferable mass spectrometry-identifiable label. Nat Commun.

[65] Yanagisawa T, Hino N, Iraha F, Mukai T, Sakamoto K, Yokoyama S (2012). Wide-range protein photo-crosslinking achieved by a genetically encoded N ε-(benzyloxycarbonyl) lysine derivative with a diazirinyl moiety. Mol BioSyst.

[66] Tomohiro T, Yamamoto A, Tatsumi Y (2013). Hatanaka Y: [3-(Trifluoromethyl)-3 H-diazirin-3-yl] coumarin as a carbene-generating photocross-linker with masked fluorogenic beacon. Chem Commun.

[67] Morimoto S, Tomohiro T, Maruyama N, Hatanaka Y (2013). Photo-affinity casting of a coumarin flag for rapid identification of ligand-binding sites within protein. Chem Commun.

[68] Tomohiro T, Morimoto S, Shima T, Chiba J, Hatanaka Y (2014). An Isotope‐Coded Fluorogenic Cross‐Linker for High‐Performance Target Identification Based on Photo-affinity Labeling. Angew Chem Int Ed.

[69] Simon B, Huang X, Ju H, Sun G, Yang M (2017). Synthesis and characterization of photo-affinity labeling reagents towards the Hsp90 C-terminal domain. Org Biomol Chem.

[70] Zhang H, Song Y, Zou Y, Ge Y, An Y, Ma Y, Zhu Z, Yang CJ (2014). A DA-based photo-affinity probe for facile and efficient aptamer–protein covalent conjugation. Chem Commun.

[71] Bai X, Lu C, Jin J, Tian S, Guo Z, Chen P, Zhai G, Zheng S, He X, Fan E (2016). Development of a DNA‐Templated Peptide Probe for Photo-affinity Labeling and Enrichment of the Histone Modification Reader Proteins. Angew Chem.

[72] Sugihara Y, Tatsumi S, Kobori A (2016). Development of novel photoresponsive oligodeoxyribonucleotides with a 2′-O-DA-conjugated adenosine for DNA interstrand cross-linking. Chem Lett.

[73] Chan EW, Chattopadhaya S, Panicker RC, Huang X, Yao SQ (2004). Developing photoactive affinity probes for proteomic profiling: hydroxamate-based probes for metalloproteases. J Am Chem Soc.

[74] Zhu B, Zhang H, Pan S, Wang C, Ge J, Lee JS, Yao SQ (2016). In Situ Proteome Profiling and Bioimaging Applications of Small‐Molecule Affinity‐Based Probes Derived From DOT1L Inhibitors. Chem A Eur J.

[75] Li Z, Hao P, Li L, Tan CY, Cheng X, Chen GY, Sze SK, Shen HM, Yao SQ (2013). Design and Synthesis of Minimalist Terminal Alkyne‐Containing DA Photo‐Crosslinkers and Their Incorporation into Kinase Inhibitors for Cell‐and Tissue‐Based Proteome Profiling. Angew Chem Int Ed.

[76] Li Z, Wang D, Li L, Pan S, Na Z, Tan CY, Yao SQ (2014). “Minimalist” cyclopropene-containing photo-cross-linkers suitable for live-cell imaging and affinity-based protein labeling. J Am Chem Soc.

[77] Jeong HS, Hayashi G, Okamoto A (2015). DA Photocrosslinking Recruits Activated FTO Demethylase Complexes for Specific N 6-methyladenosine Recognition. ACS Chem Biol.

[78] Wang L, Yoshida T, Muto Y, Murai Y, Tachrim ZP, Ishida A, Nakagawa S, Sakihama Y, Hashidoko Y, Masuda K (2015). Synthesis of DA‐Based Photoreactive Saccharin Derivatives for the Photo-affinity Labeling of Gustatory Receptors. Eur J Org Chem.

[79] Chang T-C, Adak AK, Lin T-W, Li P-J, Chen Y-J, Lai C-H, Liang C-F, Chen Y-J, Lin C-C (2016). A photo-cleavable biotin affinity tag for the facile release of a photo-crosslinked carbohydrate-binding protein. Bioorg Med Chem.

[80] Sakurai K, Yasui T, Mizuno S (2015). Comparative Analysis of the Reactivity of DA‐Based Photo-affinity Probes toward a Carbohydrate‐Binding Protein. Asian J Org Chem.

[81] Yamada R, Hiraizumi M, Narita S, Sakurai K (2016). Two‐Step Synthesis of a Clickable Photo-affinity Probe from an Anticancer Saponin OSW‐1 and its Photochemical Reactivity. Asian J Org Chem.

[82] Stewart JA, Piligian BF, Rundell SR, Swarts BM (2015). A trifunctional cyclooctyne for modifying azide-labeled biomolecules with photocrosslinking and affinity tags. Chem Commun.

[83] Chin JW, Santoro SW, Martin AB, King DS, Wang L, Schultz PG (2002). Addition of p-Azido-l-phenylalanine to the Genetic Code of Escherichia c oli. J Am Chem Soc.

[84] Mori H, Ito K (2006). Different modes of SecY-SecA interactions revealed by site-directed in vivo photo-cross-linking. Proc Natl Acad Sci.

[85] Chin JW, Cropp TA, Anderson JC, Mukherji M, Zhang Z, Schultz PG (2003). An expanded eukaryotic genetic code. Science.

[86] Liu W, Brock A, Chen S, Chen S, Schultz PG (2007). Genetic incorporation of unnatural amino acids into proteins in mammalian cells. Nat Methods.

[87] Tippmann EM, Liu W, Summerer D, Mack AV, Schultz PG (2007). A genetically encoded DA photocrosslinker in Escherichia coli. ChemBioChem.

[88] Chen S, Schultz PG, Brock A (2007). An improved system for the generation and analysis of mutant proteins containing unnatural amino acids in Saccharomyces cerevisiae. J Mol Biol.

[89] Bentin T, Hamzavi R, Salomonsson J, Roy H, Ibba M, Nielsen PE (2004). Photoreactive bicyclic amino acids as substrates for mutant Escherichia coli phenylalanyl-tRNA synthetases. J Biol Chem.

[90] Chen H-T, Warfield L, Hahn S (2007). The positions of TFIIF and TFIIE in the RNA polymerase II transcription preinitiation complex. Nat Struct Mol Biol.

[91] Hino N, Okazaki Y, Kobayashi T, Hayashi A, Sakamoto K, Yokoyama S (2005). Protein photo-cross-linking in mammalian cells by site-specific incorporation of a photoreactive amino acid. Nat Methods.

[92] Hino N, Hayashi A, Sakamoto K, Yokoyama S (2006). Site-specific incorporation of non-natural amino acids into proteins in mammalian cells with an expanded genetic code. Nat Protoc.

[93] Schlieker C, Weibezahn J, Patzelt H, Tessarz P, Strub C, Zeth K, Erbse A, Schneider-Mergener J, Chin JW, Schultz PG (2004). Substrate recognition by the AAA+ chaperone ClpB. Nat Struct Mol Biol.

[94] Weibezahn J, Tessarz P, Schlieker C, Zahn R, Maglica Z, Lee S, Zentgraf H, Weber-Ban EU, Dougan DA, Tsai FT (2004). Thermotolerance requires refolding of aggregated proteins by substrate translocation through the central pore of ClpB. Cell.

[95] Suchanek M, Radzikowska A, Thiele C (2005). Photo-leucine and photo-methionine allow identification of protein-protein interactions in living cells. Nat Methods.

[96] Li G, Liu Y, Yu X, Li X (2014). Multivalent photo-affinity probe for labeling small molecule binding proteins. Bioconjug Chem.

[97] Yang T, Liu Z, Li XD (2015). Developing DA-based chemical probes to identify histone modification ‘readers’ and ‘erasers’. Chem Sci.

[98] Horning BD, Suciu RM, Ghadiri D, Ulanovskaya O, Matthews ML, Lum KM, Backus K, Brown SJ, Rosen H, Cravatt BF (2016). Chemical proteomic profiling of human methyltransferases. J Am Chem Soc.

[99] Vervacke JS, Funk AL, Wang Y-C, Strom M, Hrycyna CA, Distefano MD (2014). DA-containing photoactivatable isoprenoid: synthesis and application in studies with isoprenylcysteine carboxyl methyltransferase. J Org Chem.

[100] Sakurai K, Yamaguchi T, Mizuno S (2016). Design and synthesis of fluorescent glycolipid photo-affinity probes and their photoreactivity. Bioorg Med Chem Lett.

[101] Sakurai K, Ozawa S, Yamada R, Yasui T, Mizuno S (2014). Comparison of the reactivity of carbohydrate photo-affinity probes with different photoreactive groups. ChemBioChem.

[102] Park J, Koh M, Koo JY, Lee S, Park SB (2015). Investigation of specific binding proteins to photo-affinity linkers for efficient deconvolution of target protein. ACS Chem Biol.

[103] YoungáKoo J, Yellamelli V, BumáPark S (2016). Nonspecific protein labeling of photo-affinity linkers correlates with their molecular shapes in living cells. Chem Commun.

[104] Liang J, Zhang L, Tan XL, Qi YK, Feng S, Deng H, Yan Y, Zheng JS, Liu L, Tian CL (2017). Chemical Synthesis of Diubiquitin‐Based Photo-affinity Probes for Selectively Profiling Ubiquitin‐Binding Proteins. Angew Chem.

[105] Muttach F, Mäsing F, Studer A, Rentmeister A (2017). Novel AdoMet analogues as tools for enzymatic transfer of photo‐crosslinkers and capturing RNA‐protein interactions. Chem A Eur J.

[106] Lin E-W, Boehnke N, Maynard HD (2014). Protein-polymer conjugation via ligand affinity and photoactivation of glutathione S-transferase. Bioconjug Chem.

[107] Bush JT, Walport LJ, McGouran JF, Leung IK, Berridge G, van Berkel SS, Basak A, Kessler BM, Schofield CJ (2013). The Ugi four-component reaction enables expedient synthesis and comparison of photo-affinity probes. Chem Sci.

[108] Kleiner P, Heydenreuter W, Stahl M, Korotkov VS, Sieber SA (2017). A Whole Proteome Inventory of Background Photocrosslinker Binding. Angew Chem Int Ed.

[109] Ramil CP, Lin Q (2014). Photoclick chemistry: a fluorogenic light-triggered in vivo ligation reaction. Curr Opin Chem Biol.

[110] Herner A, Marjanovic J, Lewandowski TM, Marin V, Patterson M, Miesbauer L, Ready D, Williams J, Vasudevan A, Lin Q (2016). 2-Aryl-5-carboxytetrazole as a New Photo-affinity Label for Drug Target Identification. J Am Chem Soc.

[111] Li Z, Qian L, Li L, Bernhammer JC, Huynh HV, Lee JS, Yao SQ (2016). Tetrazole Photoclick Chemistry: Reinvestigating Its Suitability as a Bioorthogonal Reaction and Potential Applications. Angew Chem Int Ed.

[112] Gygi SP, Rist B, Gerber SA, Turecek F, Gelb MH, Aebersold R (1999). Quantitative analysis of complex protein mixtures using isotope-coded affinity tags. Nat Biotechnol.

[113] Shiio Y, Aebersold R (2006). Quantitative proteome analysis using isotope-coded affinity tags and mass spectrometry. Nat Protoc.

[114] Thompson A, Schafer J, Kuhn K, Kienle S, Schwarz J, Schmidt G, Neumann T, Johnstone R, Mohammed AK, Hamon C (2003). Tandem mass tags: a novel quantification strategy for comparative analysis of complex protein mixtures by MS/MS. Anal Chem.

[115] Ross PL, Huang YN, Marchese JN, Williamson B, Parker K, Hattan S, Khainovski N, Pillai S, Dey S, Daniels S (2004). Multiplexed protein quantitation in Saccharomyces cerevisiae using amine-reactive isobaric tagging reagents. Mol Cell Proteomics.

[116] Raijmakers R, Berkers CR, de Jong A, Ovaa H, Heck AJ, Mohammed S (2008). Automated online sequential isotope labeling for protein quantitation applied to proteasome tissue-specific diversity. Mol Cell Proteomics.

[117] Boersema PJ, Raijmakers R, Lemeer S, Mohammed S, Heck AJ (2009). Multiplex peptide stable isotope dimethyl labeling for quantitative proteomics. Nat Protoc.

[118] Deracinois B, Flahaut C, Duban-Deweer S, Karamanos Y (2013). Comparative and Quantitative Global Proteomics Approaches: An Overview. Proteomes.

[119] Ong SE, Blagoev B, Kratchmarova I, Kristensen DB, Steen H, Pandey A, Mann M (2002). Stable isotope labeling by amino acids in cell culture, SILAC, as a simple and accurate approach to expression proteomics. Mol Cell Proteomics.

[120] Ong SE, Mann M (2006). A practical recipe for stable isotope labeling by amino acids in cell culture (SILAC). Nat Protoc.

[121] Yoo YH, Yun J, Yoon CN, Lee JS (2015). Chemical proteomic identification of T-plastin as a novel host cell response factor in HCV infection. Sci Rep.

[122] Oda Y, Huang K, Cross FR, Cowburn D, Chait BT (1999). Accurate quantitation of protein expression and site-specific phosphorylation. Proc Natl Acad Sci U S A.

[123] Lanucara F, Eyers CE (2011). Mass spectrometric-based quantitative proteomics using SILAC. Methods Enzymol.

[124] Ong SE, Kratchmarova I, Mann M (2003). Properties of 13C-substituted arginine in stable isotope labeling by amino acids in cell culture (SILAC). J Proteome Res.

